# Advances in Engineering Human Tissue Models

**DOI:** 10.3389/fbioe.2020.620962

**Published:** 2021-01-28

**Authors:** Chrysanthi-Maria Moysidou, Chiara Barberio, Róisín Meabh Owens

**Affiliations:** Department of Chemical Engineering and Biotechnology, University of Cambridge, Cambridge, United Kingdom

**Keywords:** tissue engineering, scaffold, hydrogel, 3D biology, organoid, organ-on-a-chip

## Abstract

Research in cell biology greatly relies on cell-based *in vitro* assays and models that facilitate the investigation and understanding of specific biological events and processes under different conditions. The quality of such experimental models and particularly the level at which they represent cell behavior in the native tissue, is of critical importance for our understanding of cell interactions within tissues and organs. Conventionally, *in vitro* models are based on experimental manipulation of mammalian cells, grown as monolayers on flat, two-dimensional (2D) substrates. Despite the amazing progress and discoveries achieved with flat biology models, our ability to translate biological insights has been limited, since the 2D environment does not reflect the physiological behavior of cells in real tissues. Advances in 3D cell biology and engineering have led to the development of a new generation of cell culture formats that can better recapitulate the *in vivo* microenvironment, allowing us to examine cells and their interactions in a more biomimetic context. Modern biomedical research has at its disposal novel technological approaches that promote development of more sophisticated and robust tissue engineering *in vitro* models, including scaffold- or hydrogel-based formats, organotypic cultures, and organs-on-chips. Even though such systems are necessarily simplified to capture a particular range of physiology, their ability to model specific processes of human biology is greatly valued for their potential to close the gap between conventional animal studies and human (patho-) physiology. Here, we review recent advances in 3D biomimetic cultures, focusing on the technological bricks available to develop more physiologically relevant *in vitro* models of human tissues. By highlighting applications and examples of several physiological and disease models, we identify the limitations and challenges which the field needs to address in order to more effectively incorporate synthetic biomimetic culture platforms into biomedical research.

## Introduction

Cell culture systems represent an indispensable tool for a wide range of biomedical studies. Harrison's first experiments, early in the twentieth century, on development of frog nerve fibers in a dish, the establishment of aseptic technique and subculture methods by Carrell and Ebeling in 1920s and the successful isolation and maintenance of the first immortalized human cell line (HeLa cells) by Gey in the 1950s, made it possible to grow cells in artificial environments, laying the foundation for cell and molecular biology (Taylor and Taylor, 2014; Jedrzejczak-Silicka, 2017; Simian and Bissell, 2017). Cell culture has come a long way since then and is now a vital and invaluable tool for a vast array of applications, both in academic and industrial settings, including drug development, cancer research and tissue engineering (Przyborski, 2017; Kapałczyńska et al., 2018). In such studies, two-dimensional (2D) cell culture systems dominate, continuing to improve our perception and understanding of cell biology. These cell systems rely mainly on adherent cultures, where cells grow as a monolayer attached to a plastic or glass substrate. Although easy and convenient, 2D cultures exhibit numerous disadvantages. Firstly, they are simplistic imitations of the *in vivo* situation, where cells grow within a complex three-dimensional (3D) microenvironment. The lack of this environmental context and structural architecture excludes physical cues for cell-cell and cell-matrix communication, critical for several cellular processes (e.g., mitosis, self-renewal, and differentiation). These physical constraints also impede cells from organizing naturally and spreading vertically, forcing them to flatten out and grow as monolayers (Fitzgerald et al., 2015; Przyborski, 2017). In turn, gene expression, production of proteins and cytoskeletal structure are altered, resulting in loss of the diverse cell phenotype and thus of the physiological cellular behavior and function (Birgersdotter et al., 2005; Luca et al., 2013; Fontoura et al., 2020). In addition, the absence of oxygen and nutrient gradients in monolayer cultures disrupts cell response to physiological stimuli, further inhibiting basic cellular processes and intercellular crosstalk, while the lack of a heterogeneous cell population in 2D models hinders their potential to form more complex tissue- or organ-like structures. These inherent limitations and shortcomings of 2D cell systems ultimately lead to failures in understanding cell behavior in healthy or diseased states (Duval et al., 2017). The research community is now beginning to seek alternative technologies that will facilitate development of models able to more closely mimic the complexity of whole tissues *in vitro* (Fitzgerald et al., 2015; Przyborski, 2017; Kapałczyńska et al., 2018). To this end, 3D cell cultures can provide a well-controlled *in vivo*-like microenvironment specifically tailored to each application (Chen, 2016; Koledova, 2017; Owens et al., 2017; Przyborski, 2017; Kapałczyńska et al., 2018; Jensen and Teng, 2020).

**Figure 1:**
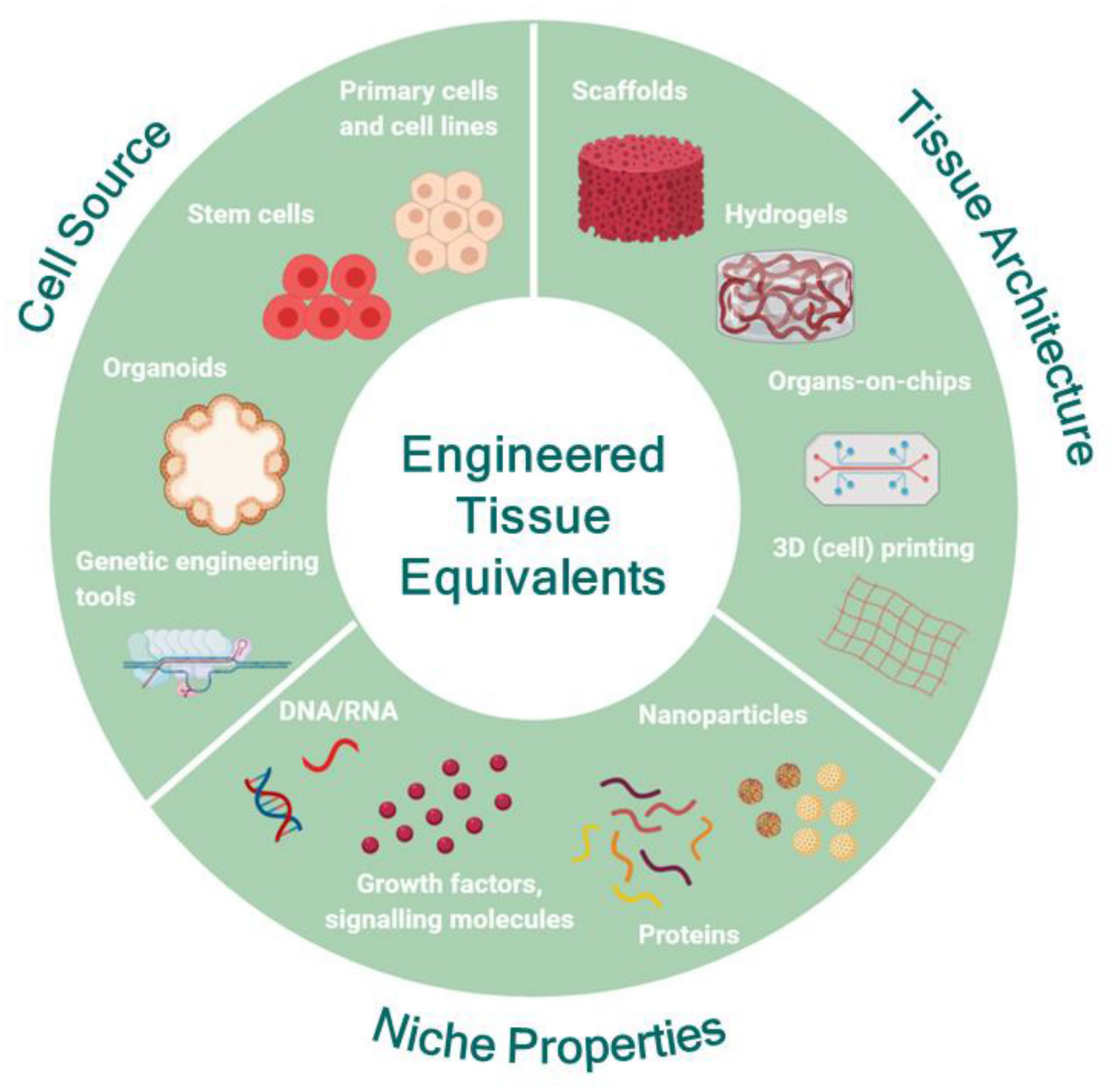
Engineering human tissue equivalents *in vitro*: the main premise for the successful development of tissue equivalents is to understand the structural and functional role of each counterpart of the native tissue and to carefully choose the range of features necessary to recapitulate the specific characteristics of the native tissue for each application. Then, the appropriate source of cells can be identified and modulated, if necessary, to capture the desired functionality. In parallel, the most appropriate substrate format can be designed and engineered to match the physicochemical properties and architecture of the native tissue under the conditions of interest and to facilitate coupling with the appropriate biochemical and biophysical cues mimicking the *in vivo* niche. Created with BioRender.com.

Although it is thought that the inception of 3D biology was in the 1970s (Schwarz and Bissell, 1977; Bissell, 1981; Bissell and Barcellos-Hoff, 1987), where cells were cultivated in floating collagen gels or agar, in fact, the phrase “three-dimensional cell culture models” was first coined in the studies of Barchelos-Hoff in 1989 and Petersen in 1992, who developed assays to distinguish between healthy and malignant breast epithelial cells grown in laminin-rich matrices (Hamburger and Salmon, 1977; Bissell, 2017; Simian and Bissell, 2017). These studies were followed by a body of research on new technologies focused on enhancing the morphological and physiological relevance of cell culture systems. The increasing number of publications since then, utilizing such cell culture platforms, or suggesting new ones, highlights the transition of the field into 3D cell culture in order to improve the capabilities of experiments performed *in vitro* (Bissell, 2017; Przyborski, 2017; Devarasetty et al., 2020). Over the years, 3D cell culture has become a generalized term, often used to point out the disparities between conventional and new cell culture technologies. Therefore, it is important to clearly define what is meant—or should be meant—by this term. Broadly speaking, we define 3D cell culture as an *in vitro* tissue-specific microenvironment that enables individual cells to grow, maintaining their 3D shape and functions, as well as to interact with their surroundings and a heterogeneous population of neighboring cells, establishing sufficient signaling networks. In this environment exogenous interference and support should be minimized (e.g., automated media perfusion) to reduce stress and unnatural cell responses and rather enable growth of different cell types to foster the development of more realistic culture systems (Abbott, 2003; Huh et al., 2011; Shamir and Ewald, 2014). 3D cell culture is also used to describe tissue- and organ-like structures emerging from the combination of 3D cell biology with Tissue Engineering (TE) principles. In these studies, researchers are focused on reconstructing organ structure and function *ex vivo* (Figure 1), to produce more reliable and physiologically relevant human-like 3D *in vitro* models (Khademhosseini and Langer, 2016; Caddeo et al., 2017), following the basic TE premise: the appropriate cell types (e.g., primary cells, stem cells) are seeded in biodegradable structures fabricated to mimic the target organ or tissue (i.e., scaffolds) and are supplied with the appropriate cocktail of substances essential for tissue generation (e.g., growth factors and signaling molecules; Langer and Vacanti, 1993). Such tissue-engineered human equivalents represent a promising alternative to the current state-of-the-art and particularly to animal models, which often fail to recapitulate human conditions due to differences in the overall physiology and in the molecular and signaling mechanisms involved in the onset and progression of diseases. This is evidenced by the high failure rates of drugs and therapies to enter clinical trials to get approval from regulatory agencies despite successful tests in animals, underlying the challenges in translating such data to human systems. Besides their greater translational relevance, the development of tissue-engineered *in vitro* models has recently taken off thanks to ethical and economic arguments (Rouwkema et al., 2011; Caddeo et al., 2017). Although the use and welfare of animals in science is protected by national and international legislation (e.g., the principle of 3Rs), there are still vibrant discussions and room for improvements as well as strong encouragement for reduction and replacement. This is also encouraged by an economic point of view, since the actual costs for drug or treatment candidates to become clinical products are huge and the process is time-consuming and labor intensive (Rouwkema et al., 2011; Fitzgerald et al., 2015).

In this review, we focus on bioengineering approaches that seek to integrate TE with 3D cell biology toward more sophisticated and reliable 3D human tissue equivalents, with the potential to (i) enhance the predictive value of preclinical studies, (ii) improve the way we study physiology and pathology and thus to address biological questions that so far necessitated animal models, and (iii) bridge the gap between current (pre-)clinical research tools and human systems by assisting and advancing drug development processes in terms of science, bioethics and economy (Rouwkema et al., 2011; Fitzgerald et al., 2015; Caddeo et al., 2017). Current trends in the field suggest that the choice of the culture format/technology/device that will support the tissue equivalent should take into account the constituent parts of the organ(s) to be modeled and the extent to which the *in vivo* complexity will be recapitulated. Various techniques and culture formats have been developed to meet these requirements, however a single format/technology/device that meets the requirements/needs of all 3D cell culture assays does not exist, and indeed should not exist, given the diversity in the morphology and functions of all the different organs/tissues researchers are emulating for different applications (Shamir and Ewald, 2014; Knight and Przyborski, 2015). Based on the format, 3D cell culture platforms can generally be categorized as scaffold-free or scaffold-based systems. Scaffold-based approaches utilize natural or synthetic materials to provide support in the form of a matrix that creates the desired tissue-specific microenvironment for optimal cell growth and differentiation and natural ECM deposition, while preserving the native tissue architecture (Fitzgerald et al., 2015; Przyborski, 2017). Decellularised scaffolds have also been used to culture cells *in vitro* by removing cells from whole tissues/organs or from the scaffold biomaterial surface after culturing cells on it for sufficient time for native ECM deposition. This way the structural and functional matrix proteins (e.g., collagen, fibronectin, hyaluronic acid, and laminin) remain intact while the exact composition varies according to the origin of tissue/organ or the cells seeded (Fitzgerald et al., 2015). Another category where cell growth and differentiation is supported by biomimetic matrices is hydrogels, which are networks of cross-linked hydrophilic polymers with the unique capability to absorb and retain copious amounts of water without dissolving but rather swelling. Similar to scaffolds, hydrogels can be made from natural or synthetic materials and cross-linked by either physical or chemical means. Due to their particularly soft nature, they are well-suited for soft tissue *in vitro* models (Fitzgerald et al., 2015; Przyborski, 2017). In contrast, scaffold-free culture systems seek the formation of multicellular masses without exogenous input as a framework, but rather by encouraging cells to form aggregates, secrete their own ECM and then self-assemble into 3D microstructures (Przyborski, 2017). This categorization however is quite generalized since the progress in, and convergence of, related disciplines have made possible the generation of new, improved, and more sophisticated tools for 3D biomimetic cultures. Currently, novel biofabrication methods (e.g., 3D printing) and microscale technologies (e.g., soft lithography), can be combined with advanced biological systems such as organoids and stem cells, resulting in more complex culture systems, tailored for specific applications. In the following sections, we first provide an overview of the current technological bricks available to develop tissue-engineered human models by summarizing the advancements in cell biology, materials science and bioengineering. Then, through examples of the current state-of-the-art, we identify and discuss the advantages, limitations and challenges the field needs to address in order for *in vitro* TE models to be successfully implemented in biomedical research.

## Building Blocks for Developing Human Tissue Equivalents

### Cell Sources for *in vitro* Tissue Engineering

To successfully design and develop tissue equivalents, it is useful to understand the anatomical and functional characteristics of the tissue of interest, as well as the role and interaction of its constituent parts (Caddeo et al., 2017). Although the end goal is to create organ and tissue equivalents with enhanced biomimicry in the lab, it is important to acknowledge the reductionist nature of these models. In fact, 3D human tissue equivalents are intentionally and necessarily reductionist, carefully designed to capture a specific range of the *in vivo* physiology over time, fit for a specific application. Therefore, a crucial step in the design process is to identify the appropriate factors that must be incorporated in order to model different *in vivo* situations (Chen, 2016). To this end, the source and the number of cells must be carefully chosen as this will determine the ability of the model to capture *in vitro* the desired characteristics of the native tissue at the cell culture level and then establish what the system might or might not recapitulate and to what extent (Chen, 2016; Caddeo et al., 2017).

#### Stem Cells

Until recently, TE approaches relied almost exclusively on established cell lines and primary cells. Despite the advantages of using cell lines (e.g., easy to use, inexpensive, unlimited availability, reproducibility, no need of ethics approval), they are not considered ideal sources for modeling human conditions since they do not exhibit normal features, often drifting from the genetic and phenotypic profile of the tissue of origin (Carter and Shieh, 2015a). Primary cells are more representative of the morphological and functional features of the tissue they are derived from, but they can be difficult to obtain and maintain for long-term experiments. Moreover, they have low proliferation rates and must be used in early passage stages because they lose their structural, functional, and self-renewal properties as they undergo senescence processes. Reproducibility of results is an additional issue when using primary cells and donor-to-donor variations must be taken into account (Benam et al., 2015; Caddeo et al., 2017).

To overcome these limitations, stem cells are now being employed for reconstructing tissue/organ structure and function *in vitro*, due to their unique capabilities to self-renew (stemness) and to differentiate toward one or more specialized cell types (potency), representing a versatile source of cellular substitutes for a wide range of applications (Avior et al., 2016; McKee and Chaudhry, 2017; Rowe and Daley, 2019). Until recently, the only source of stem cells for biomedical research was human-derived embryonic stem cell lines (ESCs). However, the discovery of human induced pluripotent stem cells (iPSCs) in 2007 (Takahashi et al., 2007) substantially altered the field of biomedical research. iPSCs are engineered stem cells generated directly from adult (differentiated) somatic cells by introducing a set of pluripotency-associated genes into cells, or through chemical reprogramming or protein delivery (Khademhosseini and Langer, 2016; Caddeo et al., 2017). These cells exhibit similar stemness and potency characteristics as ESCs and, under certain conditions and depending on their origin, they can differentiate toward various cell types. Among other applications, human iPSCs offer an unlimited supply of cells for *in vitro* TE, disease modeling, cell therapy and pharmaceutical applications. Importantly, as these stem cells can be derived from patients with specific pathology, patient-derived hiPSCs are now used to more accurately model disease and to improve diagnostics and drug discovery, laying the foundations for novel methods of personalized medicine (Cyranoski, 2018; Rowe and Daley, 2019). Despite the great potential of iPSCs to bridge the gap between preclinical studies, animal models and clinical studies, it is important to note that currently most iPSC-derived cell types exhibit immature phenotypes, while some pathologies cause such damage in adult somatic cells that the iPSCs derived from such tissues would not be informative (Benam et al., 2015; Cyranoski, 2018). Ongoing research on establishing protocols for the maturation of iPSC-derived specialized cell types will likely address these challenges via applying different biochemical and biophysical stimuli. TE strategies could be useful in these efforts by providing the appropriate microenvironment (e.g., biomimetic scaffolds) and cues (e.g., ECM rich in growth factors) to guide differentiation and maturation (Benam et al., 2015).

Finally, another type of stem cells that have gained a lot of attention as a cell source for TE are mesenchymal stem/stromal cells (MSCs), mainly due to their therapeutic potential (Rosenbaum et al., 2008; Khademhosseini and Langer, 2016). MSCs are a specific subtype of multipotent stem cells, diversely distributed in the human body including bone marrow, adipose, perinatal tissues, blood, periodontal ligament, and skeletal muscles, from most of which they can be isolated (Rosenbaum et al., 2008; McKee and Chaudhry, 2017; Ullah et al., 2019). Despite having the same capabilities as pluripotent stem cells, MSCs can differentiate toward only a few specific cell types, such as osteogenic, chondrogenic, and adipogenic cell types, depending on the nature and maturity of the tissue of origin (Rosenbaum et al., 2008; McKee and Chaudhry, 2017). Their homing capability has made them very attractive candidates for a wide breadth of preclinical and clinical applications, including tissue regeneration, wound healing, and treatment of autoimmune diseases (Khademhosseini and Langer, 2016; Ullah et al., 2019). Upon injury, MSCs are naturally released in the circulation and migrate to the damaged tissue where they secrete a pool of cytokines, growth factors and other bioactive molecules with immunomodulatory and angiogenic effects, thus creating a microenvironment that promotes tissue repair and regeneration (Ullah et al., 2019). For *in vitro* TE, MSCs represent a useful resource, mainly due to their ease in isolation, manipulation and differentiation, compared to the longer and more elaborate iPSCs protocols.

#### Organoids

The advent of human pluripotent stem cells marked the starting point of the development of “organs in a dish,” also known as organotypic cultures or organoids, a major breakthrough of the past decade (Dutta et al., 2017; Lancaster and Huch, 2019). The term “organoid” is not new; it was used in the 1950s and 1960s to describe structures in cell culture systems that resembled organs (Duryee and Doherty, 1954; Schneider et al., 1963; Wolter, 1967) and more recently in studies where 3D cell aggregates, called spheroids, were defined as organoids despite the fact that they were not fully representative of the native tissue (Dutta et al., 2017). The term has become popularized in *in vitro* biology and evolved to generally refer to tissues or structures that resemble an organ, losing its precision (Dutta et al., 2017; Lancaster and Huch, 2019). A more specific working definition that fulfills the basic definition of organoids was recently proposed, along with several criteria: “(1) a 3D structure containing cells that establish or retain the identity of the organ being modeled; (2) the presence of multiple cell types, as in the organ itself; (3) the tissue exhibits some aspect of the specialized function of the organ; and (4) self-organization according to the same intrinsic organizing principles as in the organ itself” (Lancaster and Huch, 2019). These properties render organoids suitable formats/tools for modeling organ architecture *in vitro*. Because iPSC-derived organoids follow *in vivo* like development, their morphology closely recapitulates the native organ structure, making them particularly apt for studies looking at developmental organogenesis, while tissue-specific adult stem cell organoids are mostly suited for studying tissue homeostasis and maintenance, since naturally in the body they are key players in these processes (Fatehullah et al., 2016; Yin et al., 2016; Lancaster and Huch, 2019).

Currently, several protocols exist for the development of organoids for various organs, derived either from pluripotent stem cells (ESCs and iPSCs) or from organ-specific adult stem cells (ASCs) and progenitor cells (Takebe and Wells, 2019). The establishment of a protocol for a long-term, well-defined, and stable culture of murine intestinal organoids in 2009 by Sato et al. (2009) and subsequent adaptation of the protocol and modifications of the growth factor cocktail in the original organoid culture medium allowed the generation of human organoids from various tissues, such as stomach (Bartfeld et al., 2015), liver (Takebe et al., 2013), esophagus (Li et al., 2018), lung (Dye et al., 2015), and ovaries (Kessler et al., 2015; see Kim et al., 2020, for an extended review). Organoids now represent a powerful tool for a wide spectrum of biomedical applications ranging from basic cell biology studies, organogenesis and tissue homeostasis to disease modeling (see Lancaster and Huch, 2019, for an extended review), drug/therapy development, and regenerative medicine (Schweiger and Jensen, 2016; Brassard and Lutolf, 2019). However, there are several general shortcomings and challenges in the development and application of organoids, as well as in the interpretation and translation of the derived data. An important issue is the reproducibility and consistency of organoids from batch to batch. The initial culture conditions and the environment in which organoids grow are of paramount importance for their self-organization and the development of the desired emergent tissue (Brassard and Lutolf, 2019). To date, most organoid systems rely on animal-derived ECMs, such as Matrigel, supplemented with growth factors and endogenous signaling molecules (e.g., Wnt, Noggin, and R-spondin). Even though Matrigel works as an artificial niche, mimicking the native tissue environmental cues, its poorly understood composition, heterogenous nature, and batch-to-batch variability hinders the reproducibility and robustness of the organoid systems, often leading to heterogeneity in size, shape, and viability, even between organoids in the same culture (Fatehullah et al., 2016; De Souza, 2018; Brassard and Lutolf, 2019; Lancaster and Huch, 2019; Kim et al., 2020). In addition, although these organotypic cultures are highly biologically relevant, they alone do not necessarily recapitulate the dynamics present in the human system. For example, the majority of organoid culture systems lack essential components of their living counterparts, such as the enteric nervous system, the immune system, as well as luminal flow and peristalsis (In et al., 2016; Tsakmaki et al., 2017; De Souza, 2018; Kim et al., 2020). Also, lack of vascularization in organoid cultures means that their growth and development depends on diffusion of nutrients from the surrounding media. While this might not be an issue for small organoids, in cases of some larger organoids the diffusion of nutrients is limited, resulting in dramatic necrosis in their interior and hence compromising the long-term viability of the system and the validity of the results (McMurtrey, 2016; Grebenyuk and Ranga, 2019; Lancaster and Huch, 2019). To overcome this limitation, culturing organoids under flow within microfluidic chips has been proposed recently, as a means to induce vascularization and hence to improve the morphological and functional characteristics of the bioengineered tissues (Homan et al., 2019).

The tremendous potential of organoid culture systems could not go unnoticed by tissue engineers. Firstly, since organoid systems are designed and developed to recapitulate the environment and properties of the stem cell niche and the tissue progeny with indefinite culture potential, they represent an alternative, more accessible, and scalable source for harnessing stem cells (Yin et al., 2016). Employment of molecular technology and organoids in the service of *in vitro* TE can further enhance the potential of these systems in mimicking the *in vivo* conditions. In particular, genes within organoids can be manipulated using tools, such as CRISPR/Cas-9, to either correct mutations and restore physiological function or to introduce mutations and model various disease phenotypes (Dutta et al., 2017; Lancaster and Huch, 2019; Kim et al., 2020), again providing an alternative source of cells with broad applicability and amenable to manipulation. Additionally, since organoids may contain more than one tissue representative cell type, they can be used as a single cellular input for tissue-engineered equivalents, allowing them to better capture the cellular diversity of the living counterparts (Kasendra et al., 2018).

Stem cells and organoids are a versatile source of cells for *in vitro* TE applications, thus the convergence of these fields can be mutually beneficial. On one hand the use of human-derived cellular parts enhances the relevance of tissue-engineered equivalents, both in terms of mimicry and data interpretation. On the other hand, tissue engineering provides a broad toolbox to study stem cells and organoids and to address challenges related to engineering the appropriate niche for controlling the culture conditions. It also provides the extrinsic instruction patterns to robustly and elaborately direct self-organization processes. This way TE approaches can aid stem cells and organoids in realizing their full potential as *in vitro* tools for biomedical research (Benam et al., 2015; Chen, 2016; McKee and Chaudhry, 2017; Brassard and Lutolf, 2019).

### Materials for *in vitro* Tissue Engineering

**Figure 2:**
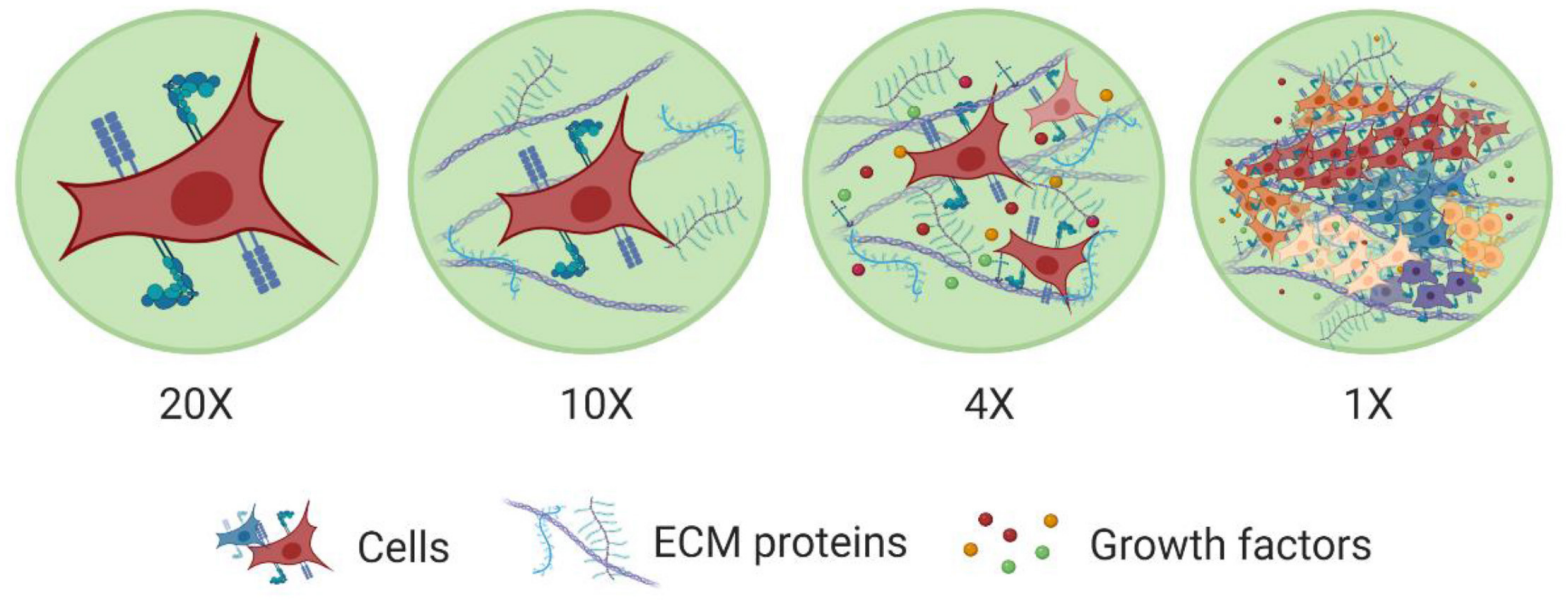
Levels of organization within tissues. The major building block of tissues are cells. Cell membranes are equipped with a wide range of proteins that help them sense and respond to cues in their microenvironment. Cells also interact with other cells and the surrounding ECM network. This ECM network comprises of various macromolecules (e.g., proteins, polysaccharides) and soluble factors (e.g., growth factors) immobilized in its structure, which promote cell-cell and cell-ECM interactions. This way ECM establishes a favorable niche for cells to grow, spread, differentiate, and perform various functions and also to work together with other cells to form more complex structures toward tissues with specific functionalities. Created with BioRender.com.

**Figure 3:**
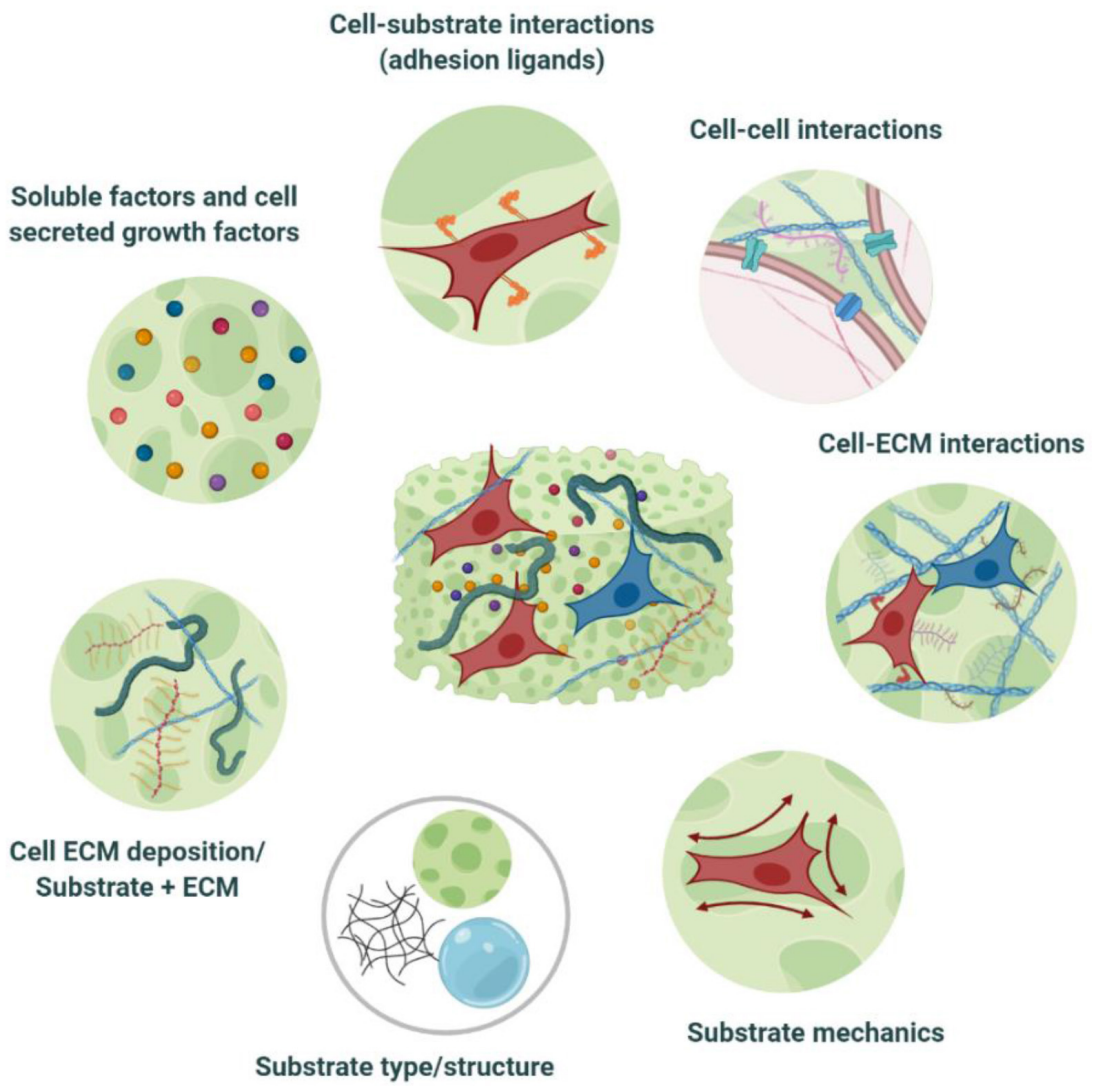
Cell-biomaterial interactions within tissue engineered equivalents. TE substrates constitute an artificial ECM, encompassing *in vivo-*like, tissue-specific biochemical, and biophysical cues. This niche provides a favorable microenvironment for cells to adhere, proliferate, differentiate, maturate, and deposit their own ECM, as well as to communicate and to establish the necessary cell-cell, and cell-ECM interactions that will enable them to auto-organize as they would *in vivo*. Modulation of the niche properties can also guide cell bahaviour toward the desired phenotypic output. Created with BioRender.com.

Alongside the appropriate cell type(s), another fundamental element for effectively engineering tissue equivalents is the choice of the suitable biomaterial(s). In the classic TE paradigm, pre-engineered 3D supports/scaffolds, made from natural or synthetic materials, are used as templates for cell attachment, growth and differentiation toward functional living constructs (Vacanti, 2006). As scaffolds act as a synthetic ECM, modifying the biomaterial building blocks to mimic the native tissue ECM is a major challenge. In the body, ECM is a 3D network that consists of various macromolecules, including proteins and polysaccharides, responsible for tissue support and maintenance, cell-cell and cell-ECM communication, diffusion of nutrients metabolites and growth factors. In addition, ECM mediates signaling pathways from soluble factors and other sources, regulating various cellular behaviors, such as migration, adhesion, proliferation, and differentiation (Frantz et al., 2010; Theocharis et al., 2016; Afewerki et al., 2019; Figure 2). In this context, the role of biomaterials for tissue engineered equivalents is to provide cells with the appropriate framework/template to adhere, proliferate, differentiate, maturate, secrete ECM and form the necessary cell-cell, and cell-scaffold interactions that will enable them to auto-organize as they would *in vivo* (Przyborski, 2017; Afewerki et al., 2019; Figure 3). The choice of biomaterial depends on the application and the physiological or pathophysiological conditions the tissue model aims to emulate (Caddeo et al., 2017). However, there are many other properties that need to be considered when selecting biomaterials, including biocompatibility, strength and elasticity, porosity, molecular gradients and mass transport of nutrients and growth factors, oxygenation, adhesion or signaling sites, surface roughness, shape, type (e.g., scaffold or hydrogel), and source (Sitarski et al., 2018), in order to more realistically recapitulate the cues naturally occurring in the native tissue.

A broad range of materials is now available for the fabrication of various types of substrates, the properties of which can be tailored at the micro- and nano-scale to match the requirements of specific applications (Huang et al., 2017; Nikolova and Chavali, 2019; Cembran et al., 2020). A characteristic property of TE scaffolds that particularly affects primary cell-matrix interactions as well as cell behavior and fate upon seeding, is its topography. It is well-known that micro-topography (1–100 μm) is responsible for cell recruitment, adhesion, orientation and gene expression, while the submicron and nano- features strongly influence the cytoskeletal arrangement (Hayes and Richards, 2010). Tailoring surface topography of biomaterials has been shown to support and enhance differentiation of MSCs toward specific lineages (Abagnale et al., 2015). Mechanobiology studies have revealed that, amongst other cues, cells are also responsive to the material stiffness (Discher et al., 2005), which can affect intracellular signaling cascades that trigger cell adhesion, phenotype maintenance, cytoskeletal reconstruction, and even stem cell differentiation (Engler et al., 2006; Mao et al., 2016; Cao et al., 2017; Kumar et al., 2017; Vining and Mooney, 2017; Darnell et al., 2018). Therefore, selection of biomaterials with the appropriate stiffness is not only important for matching the native tissue mechanical properties, but can be also used as a tool to control cell phenotypes and thus modulate cell behavior (Khademhosseini and Langer, 2016; Ledo et al., 2020b). Furthermore, materials can undergo several chemical modifications, to improve their physicochemical properties as well as to enable incorporation of biologically relevant molecules and signals necessary for guiding and regulating cell response. For example, proteins from the ECM (e.g., hyaluronic acid, collagen, fibronectin etc.) can be blended or grafted to the material surface to improve cell behavior, by acting as matrix-associated biological cues, regulating cell attachment (e.g., via integrin-mediated binding), as well as proliferation and infiltration within the scaffold (Baker and Chen, 2012). For instance, incorporation of well-known cell binding motifs from ECM-derived proteins, such as RGD (arginine-glycine-aspartate) peptide, can enhance cell spreading, and viability in hydrogels (Gallagher et al., 2020).

Biomaterials may be prepared of natural polymers such as collagen, laminin, and hyaluronic acid, or from synthetic materials such as polyethylene glycol (PEG), propylene glycol diacetate (PGDA), polyvinylidene fluoride (PVDF), or co-polymers (Afewerki et al., 2019; Nikolova and Chavali, 2019). Thus, they can be categorized as natural, synthetic, or hybrid biomaterials (see Table 1 for examples of biomaterials and applications). In most cases, naturally derived biomaterials are amino acid-based or sugar-based biopolymers which can be components of the natural ECM (e.g., collagen, laminin, elastin, and fibrinogen) or not (e.g., chitin, silk fibroin, chitosan, and alginate; Silva et al., 2017; Ahadian et al., 2018). Such materials represent an attractive source for *in vitro* TE applications, due to their microstructure, stability, biocompatibility, and ability to present cells with natural adhesion sites, as well as due to the possibility to tailor and control their properties via physical or chemical treatments (i.e., cross-linking) or by blending them with other biopolymers (Guarino et al., 2016; Ullah and Chen, 2020) to better recapitulate *in vitro* the physiological milieu. A commonly used natural biomaterial in 3D biomimetic cultures and tissues is collagen, as it is a major component of the natural ECM and it is among the main structural proteins of most connective tissues (Ahadian et al., 2018; Sorushanova et al., 2019). The prevalence of collagen in human tissues, its excellent properties (e.g., low immunogenicity, biocompatibility, biodegradability, hydrophilicity, easy processing, good encapsulation response, etc.; Ahadian et al., 2018; Liu X. et al., 2019) and the advances in preparation and cross-linking methods to boost its physicochemical properties have enabled the fabrication of various types of collagen-based bioactive substrates (e.g., scaffolds, gels, fibers, and sponges; Uchino et al., 2009; Yip and Cho, 2013; Patel et al., 2019; Sorushanova et al., 2019; Ferro et al., 2020). Skin substitute studies, for instance, have extensively employed this natural material for wound healing purposes (Min et al., 2018), while collagen hydrogels have also been shown to effectively support and enhance the growth and survival of primary cortical neurons in a 3D mimetic environment (Evans et al., 2019).

**Table 1:** Examples of biomaterials, fabrication methods and cell sources for skin, brain, heart, lung, intestine, bone, and liver biomimetic cultures.

**Material(s) and format**	**Fabrication method**	**Cell source**	**References**
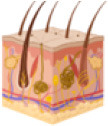 **Skin biomimetic cultures**			
Collagen hydrogel	Gelation/3D bioprinting	Fibroblasts, melanocytes (MCs), and keratinocytes (KCs)	Min et al., 2018
		Fibroblasts and KCs	Lee et al., 2009
Alginate/Carboxymethyl cellulose/Nanofibrillated cellulose (ALG/CMC/NFC) bioinks		Human skin fibroblasts (hSF)	Zidarič et al., 2020
Collagen I scaffolds	Gelation/vitrification	Normal human skin fibroblasts (NhSF), Normal human dendritic cells (NHDC), Normal human epidermal keratinocytes (NHEK)	Uchino et al., 2009
Silk Fibroin/Collagen (SF/COL) scaffolds	Freeze-drying	Primary neonatal foreskin fibroblast (NH), hiNSCs	Vidal et al., 2019
Polycaprolactone (PCL), Polycaprolactone/Collagen (PCL/COL), Polycaprolactone/Poly (L-lactic acid) (PCL/PLLA), Polycaprolactone/Poly (L-lactic acid)/Collagen (PCL/PLLA/COL) scaffolds		MSCs	Rahmani et al., 2018
Polycaprolactone/Aloe vera (PCL/AV), Polycaprolactone/Curcumin (PCL/CUR), Polycaprolactone/Aloe Vera/Tetracycline hydrochloride (PCL/AV/TCH) scaffolds	Electrospinning	Human dermal fibroblasts	Ezhilarasu et al., 2019
Gelatin methacrylate/Nanofibrils (GelMa/NF), Gelatin/Nanofibrils			Rnjak et al., 2009
Milk protein/Polycaprolactone (MP/PCL) scaffolds		Human keratinocytes	Hewitt et al., 2019
Synthetic Elastin (SE) hydrogels		Human dermal fibroblasts, Human keratinocytes	Mao et al., 2018
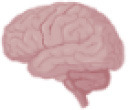 **Brain biomimetic cultures**			
Collagen gels	Gelation	Neuroblastoma cell line (SK-N-BE)	Villasante et al., 2017
Alginate/Collagen hydrogels		hiPSCs	Moxon et al., 2019
Gelatin hydrogels coated with Collagen IV/Fibronectin		hiPSC, human brain microvascular endothelial cells (BMECs)	Faley et al., 2019
Silk fibroin scaffold/Collagen I hydrogels	Silk extraction/Salt-leaching	hiPSCs	Rouleau et al., 2020
Liquid crystal elastomers (LCE) scaffolds	Salt-leaching	SH-SY5Y	Prévô et al., 2018
Silk fibroin (SF) scaffolds	Freeze-drying	hiNSCs	Cairns et al., 2020
Pol(vinyl alcohol)/Sodium alginate (PVA/SA) fibers	Multilayer Coaxial Laminar Flow	hiPSCs	Zhu et al., 2017
Sodium Alginate/Gelatin (SA/Gel) based bioinks	Gelation/3D bioprinting	SH-SY5Y, hiPSCs	Fantini et al., 2019
Gelatin Methacrylate (GelMa), Glycidyl/Methacrylate/Hyaluronic acid (GM/HA) hydrogels		Neural Stem Cells (NSCs)	Tang et al., 2020
Methacrylated Alginate (AlgMA) hydrogels		Neuroblastoma cell line (SK-N-BE)	Monferrer et al., 2020
Poly(desaminotyrosyl tyrosine ethyl ester carbonate) (pDTEc) nanofibers	Electrospinning	Neural reprogrammed stem cells (RN-iPS)	Carlson et al., 2016
Polycaprolactone (PCL) scaffolds		Human neural progenitor stem cells (hNPCs)	Jakobsson et al., 2017
Polyethylene diacrylate (PEGDA) scaffolds	UV polymerization		Murphy et al., 2020
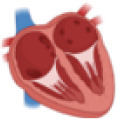 **Heart biomimetic cultures**			
Collagen nanofibers	Electrospinning	Human Bone marrow mesenchymal stem cells (hBM-MSC)	Joshi et al., 2018
Poly(vinylidene fluoride)/Trifluoroethylene (PVDF/TrFE) scaffolds		hiPSCs, Cardiomyocytes (CMs)	Adadi et al., 2020
Collagen fibers	Melt electro-wiring	Human umbilical cord vein smooth muscle cells (HUVSMCs)	Saidy et al., 2019
Alginate (Alg) hydrogel nanofibers, Alginate/Gelatin (Alg/GelF/MA) hydrogel nanofibers	Wet-electrospinning	Mesenchymal stem cells enhanced with enhanced green fluorescent protein (hEGFP-MSCs)	Majidi et al., 2018
Gelatin hydrogels	Gelation/3D bioprinting	hMSCs	Tijore et al., 2020
Hyaluronic Acid/Arginin-Glycine-Aspartic Acid (HA/RGD) hydrogels			Gallagher et al., 2020
Gelatin Methacrylate (GelMa) bioinks		HUVECs, CMs	Zhang et al., 2016b
Gelatin Methacrylate/Polyethylene diacrylate (GelMa-PEGDA) bioinks		Induced multipotent stem cells (iMSCSs)	Nachlas et al., 2020
Gelatin/Gellan Gum (GG) hydrogels		hiPSC-CMs	Koivisto et al., 2019
Poly(vinyl alcohol) (PVA) scaffolds	Freeze-drying		Dattola et al., 2019
Polycaprolactone films (MacPCL)	Layer-by-layer assembly, Laser perforation	hMSCs	Zhang et al., 2019
Matrigel coated fiber matrices	Two-photon polymerization	hiPSC-CMs	Wang C. et al., 2020
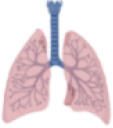 **Lung biomimetic cultures**			
Collagen hyaluronate (CHyA-B) scaffolds	Freeze-drying	Bronchial epithelium cells (Calu-3)	O'Leary et al., 2016
Poly(L-lactide-co-glycolide)/Gelatin (PLGA/Gel), Poly(L-lactide-co-glycolide)/Sodium bicarbonate (PLGA-SBC) microparticles		Lung adenocarcinoma cells (A549)	Kuriakose et al., 2019
Hyaluronic Acid-Furan/ Modified methylcellulose with reactive thiols (HA-Furan/MC-SH) hydrogels	Gelation	Smooth muscle cells (SMCs)	Tam et al., 2019
Poly(vinyl chloride)(PVC) sheets		Lung adenocarcinoma cells (A549)	Simon et al., 2016
Polyethylene terephthalate (PET) nanofibers	Electrospinning	Human airway smooth muscle (HASM)	Morris et al., 2014
Polyethylene terephthalate (PET) scaffolds		Lung fibroblasts (MRC5)	Htwe et al., 2015
Polyurethanes/polyhedral oligomeric silsesquioxane (PU/POSS) scaffolds	3D bioprinting	Human bone marrow mesenchymal stem cells (hBM-MSCs)	Wu et al., 2020
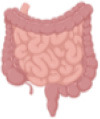 **Intestine biomimetic cultures**			
Collagen scaffolds	Gelation	Caco-2	Kim et al., 2014
Propylacrylamide (pNIPAM) hydrogels		Caco-2, HT29-MTX, hiNSCs	Dosh et al., 2017
Silk fibroin (SF) scaffolds	Freeze-drying		Shaban et al., 2018
			Manousiouthakis et al., 2019
		Human colonoid culture	Roh et al., 2019
Collagen scaffolds	Curing/gelation	Caco-2	Yu et al., 2012
Polyethylene diacrylate (PEGDA)/Acrylic acid/Fibronectin and composite scaffolds	3D bioprinting		Creff et al., 2019
Polyethylene diacrylate/Alginate acid (PEGDA/AA) scaffolds	UV photo-polymerization		Castaño et al., 2019
Polyethylene terephthalate (PET) nanofibrous scaffolds	Electrospinning		Patient et al., 2019
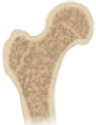 **Bone biomimetic cultures**			
Ulvan/gelatin (UG) scaffolds	Gelation/Freeze-drying	Human adipose-derived mesenchymal stem cells (hADMSCs)	Tziveleka et al., 2020
Alginate/Gelatin (Alg/Gel) scaffolds	3D bioprinting	hMSCs	Zhang et al., 2020
Polycaprolactone/Calcium-polyphosphate (PCL/Ca-polyP) microspheres		Human osteoblast-like cells (SAOS-2)	Neufurth et al., 2017
Alginate/Gelatin (Alg/Gel) scaffolds coated with graphene oxide (GO)		hADSCs	Li et al., 2020
Polycaprolactone/Poly(L-lactic acid)/Hyaluronic Acid (PLA/PCL/HA) scaffolds		Human osteocarcinoma cell line (MG63)	Hassanajili et al., 2019
Gelatin Methacrylate (GelMA)-VEGF hydrogels		MSCs	Byambaa et al., 2017
Keratin sponges	Casting	Human osteoblast-like cells (SAOS-2)	Bloise et al., 2020
Poly(L-lactic acid)/Dimethyl sulphoxide (PCL/DMSO) scaffolds	3-Dimensional plotting system (3DPS)	Human bone marrow stromal cells (hBMSCs)	Seok et al., 2020
Poly(L-lactic acid) (PCL), Poly(L-lactic acid)/Silicate-containing hydroxyapatite (PCL-siHA) scaffolds	Electrospinning	hMSCs	Shkarina et al., 2018
Poly(3,4- ethylene dioxythiophene)/Collagen (PEDOT/COLL) scaffolds	Freeze-drying	Neural crest stem cells (NCSCs)	Iandolo et al., 2020
Polypyrrole crosslinked (PPY/XCS) scaffolds		hADMSCs /HUVECs	Zhang et al., 2018
Tricalcium phosphate/Alginic acid/Graphene Oxide (TCP/AA/GO) scaffolds	Polymerization/3D bioprinting	Human osteoblast cells (hOB)	Boga et al., 2018
Magnesium-β-Tricalcium Phosphate Composite (Mg-TCP) scaffolds	Gelation/ 3D bioprinting	hMSCs, HUVECs	Gu et al., 2019
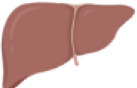 **Liver biomimetic cultures**			
Collagen gels	Gelation	Human hepatocarcinoma cells (HepG2)	Yip and Cho, 2013
Glycyrrhizin /Alginate/Calcium (GL/Alg/Ca) hydrogels	Gelation/Freeze-drying		Tong et al., 2018
Chitosan/Gelatin (CS/Gel) scaffolds			Zhang et al., 2016c
	Bioprinting/Gelation/ Freeze-drying		Gong et al., 2014
Decellularized extracellular matrix (dECM)based hydrogels	3D bioprinting		Ma et al., 2018
Gelatin Methacrylate (GelMA) hydrogels		Human hepatocarcinoma cells (HepG2/C3A)- HUVECs	Massa et al., 2017
Gelatin Methacrylate/Decellularized extracellular matrix (GelMa/dECM) bioinks		Human induced hepatocytes (hiHep)	Mao et al., 2020
Collagen/Hyaluronic Acid (COL/HA) bioinks		Activated hepatic stellate cells (aHSC)	Mazzocchi et al., 2019
Poly(L-lactide-co-glycolide)/Collagen I (PLGA/COL I) nanofibrous scaffolds	Electrospinning	Primary human hepatocytes	Brown et al., 2018
Poly(ethylene glycol)/Alginate/Gelatin (PAG) cryogel matrices	Cryogelation	Human hepatocarcinoma (Huh-7), HepG2	Kumari et al., 2016
Inverted colloidal crystal (ICC) hydrogel scaffolds	UV photo-polymerization	Huh-7	Kim M. H. et al., 2016
Polycaprolactone (PCL) micro-scaffolds	Selective Laser Sintering	HepG2, HUVECs	Pang et al., 2020

*Created with BioRender.com*.

Blends of natural materials with other biomolecules or synthetic polymers are also commonly used for the recapitulation of the tissue milieu, as a means to overcome drawbacks related with the poor mechanical properties of some natural polymers [e.g., collagen (Ullah et al., 2018), gelatin (Han et al., 2014)], the low solubility in water [e.g., keratin (Wang et al., 2016)], their limited biostability (Pedron et al., 2013; Ryan and O'Brien, 2015), as well as source availability, and their uncontrollable biodegradation (Pradhan et al., 2020). In fact, such blends allow for enhancement of the mechanical properties and for better control over the biochemical properties of the engineered substrates according to the requirements of the tissue under development. For example, addition of elastin to porous collagen scaffolds was shown to reduce stiffness and enhance viscoelasticity, while inducing a more contractile-like smooth muscle cell phenotype (Ryan and O'Brien, 2015). Combination of collagen with HA was shown recently to yield a bioink/hydrogel suitable for 3D printing liver tissue constructs containing primary human hepatocytes and liver stellate cells, that were viable and functional for over 2 weeks and able to respond to drugs (Mazzocchi et al., 2019). In addition, PLGA nanofibrous scaffolds treated with type I collagen or fibronectin, as the minimal essential ECM components of the liver microenvironment, were able to accommodate long-term *in vitro* support, maintenance, and function of primary human hepatocytes (Brown et al., 2018).

Another commonly used material is gelatin-polysaccharide composite hydrogels (Afewerki et al., 2019). The chemical similarities of gelatin to the native tissue ECM, its biocompatibility, low antigenicity, cost-effectiveness, and combination with polysaccharides have been shown to produce composite hydrogels with enhanced ECM biomimicry levels, increased mechanical resilience (Afewerki et al., 2019); hydrophilicity (Jansen et al., 2005); and antimicrobial and anti-inflammatory properties (Wang et al., 2007), thus highly promising materials for 3D cell culture and TE applications (Afewerki et al., 2019). This was exemplified by the study of Guan et al., who showed that porous gelatin-chitosan scaffolds, loaded with hyaluronic acid and heparan sulfate, offer a valuable option for neural tissue engineering as they form a suitable 3D microenvironment for the adhesion, growth and differentiation of neural stem and progenitor cells (Guan et al., 2013).

### Engineering Methods to Reconstitute Tissue Architecture *in vitro*

The choice of the most suitable biomaterial is coupled with the fabrication method, as this can also influence the final properties of the 3D matrix supporting the engineered tissue (Mabrouk et al., 2020). Various fabrication methods have been utilized so far, spanning from freeze-drying (Mabrouk et al., 2020) and physical/chemical cross-linking reactions (Hu et al., 2019), for scaffold and hydrogel preparation, respectively, to microscale technologies, such as soft-lithography for microfluidic channel fabrication (Khademhosseini et al., 2006).

**Figure 4:**
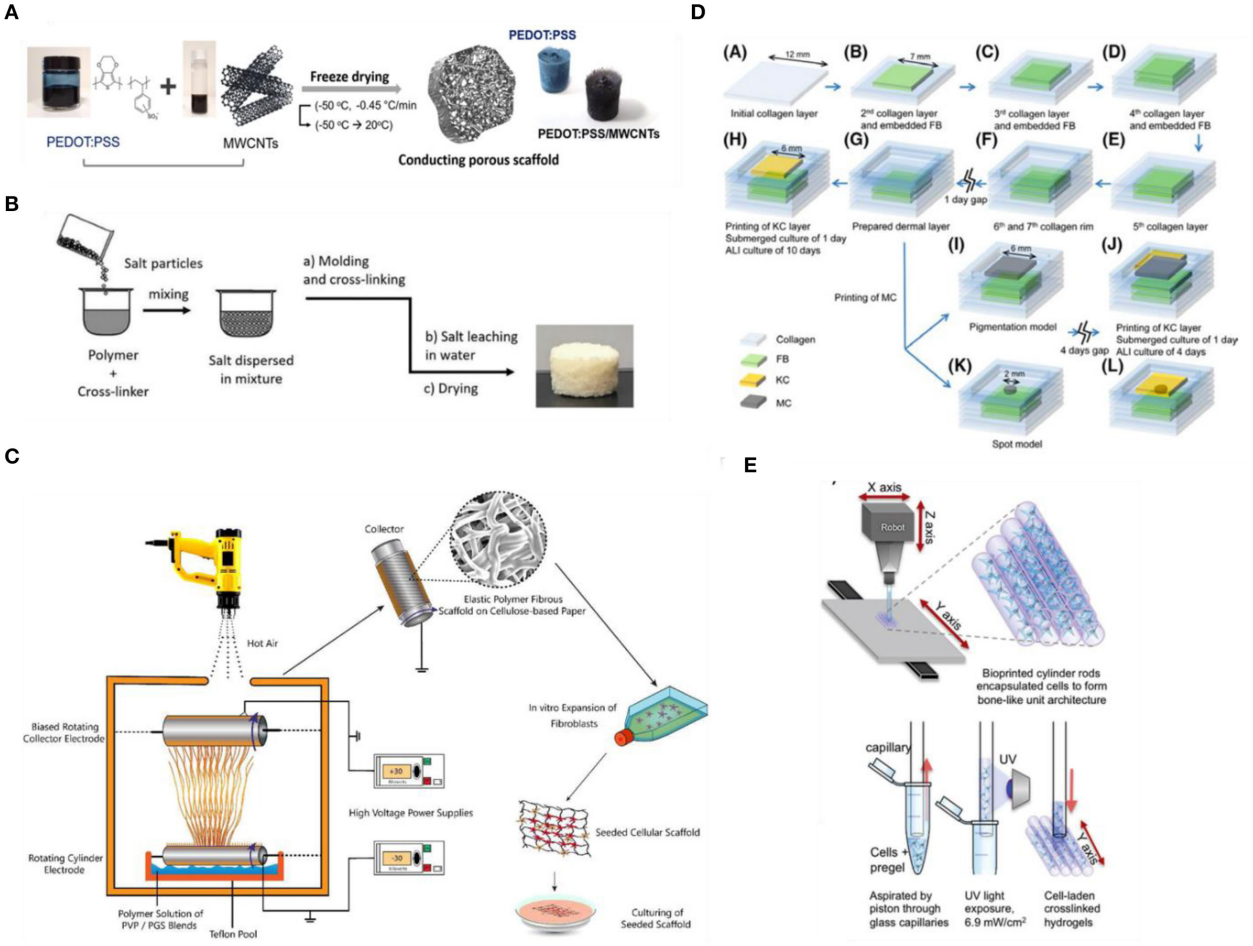
Fabrication methods for engineering 3D scaffold-based biomimetic models *in vitro*. **(A)** Fabrication process of 3D hybrid scaffolds based on PEDOT:PSS and multi-walled carbon nanotubes (MWCNT) composites via freeze-drying. Reproduced from Jayaram et al. (2019) under the Creative Commons Attribution license. **(B)** Preparation of porous Liquid Crystal Elastomers (LCE), as biodegradable brain tissue scaffolds, via salt-leaching. Reproduced from Prévô et al. (2018). **(C)** Electrospinning of Polyvinylpyrrolidone/Poly(glycerol sebacate) fibrous scaffolds for skin tissue engineering. Reproduced from Keirouz et al. (2019). 3DP techniques for engineering **(D)** skin and **(E)** bone tissue equivalents. Reproduced from Byambaa et al. (2017) and Min et al. (2018), respectively.

Conventional scaffold fabrication approaches have relied on techniques such as freeze-drying (Figure 4A), solvent casting/particulate leaching (SCPL) (Figure 4B), melt molding and gas foaming with which different porosity levels can be achieved (Wang et al., 2006). Due to the simplicity of established protocols and the relatively low-cost and tunability of scaffold porosity and geometry, these techniques are the standard for fabricating scaffolds (Mabrouk et al., 2020). However, several drawbacks have been reported, including cases of low interconnectivity of the porous network, irregular pore sizes, use of organic solvents with possible toxic effects and lack of precise control over the overall mechanical properties (El-Kady et al., 2012; Hribar et al., 2014). In an attempt to overcome these limitations, several groups employed electrospinning (Figure 4C) to fabricate various 3D matrices from both natural or synthetic materials and blends, as this method has been shown to provide better control over the mechanical properties (e.g., porosity and tensile strength), the geometry and the micro- and nano-topography of the fibrous scaffolds (Cui et al., 2010). Htwe et al. (2015) fabricated electrospun polyethylene terephthalate (PET)-based nanofibrous scaffolds, with similar geometry to human lung extracellular matrix, to form 3D cultures of lung fibroblasts as a biologically relevant tool for the investigation of such cells in the pathogenesis of lung inflammation via activating the NF-κB signaling pathway (Htwe et al., 2015). In addition, electrospun PET-based 3D nanofibrous scaffolds, coated with collagen and mimicking the basement membrane structure, were shown to successfully support an *in vitro* model of the human intestinal barrier that exhibited superior performance as a drug-testing platform compared to conventional models (Patient et al., 2019).

Over the last few years, additive manufacturing (AD) approaches have also gained a lot of attention as an alternative fabrication route to organize cells in 3D due to their potential to provide precise spatiotemporal control over biophysical and biochemical cues necessary to reproduce a biomimetic microenvironment (Murr, 2016; Bose et al., 2020; Nikolaev et al., 2020; Sun et al., 2020). AD approaches include techniques such as three-dimensional printing (3DP) (Figures 4D,E), light-assisted bioprinting (Trautmann et al., 2018), fused deposition modeling (FDM), selective laser sintering (SLS), that, along with advances in biomaterials and bioinks, enable precise deposition of materials into custom shapes and patterns to replicate complex tissue architectures, not possible using conventional techniques, and with high control and reproducibility (Melchels et al., 2012; Cui et al., 2017). Moreover, AD approaches, and specifically 3DP techniques, based on coupling multimaterial printing with high performance bioinks (i.e., hydrogel solutions that act both as cell carriers, and structural components to control and direct cell activity and fate; Chimene et al., 2020) and biomolecules have been developed to obtain highly customisable, biofunctional, and mechanically compliant scaffolds (Chimene et al., 2016). The potential of bioprinting for building such biorelevant models is highlighted in various recent studies, seeking to develop highly biomimetic and functional tissues for disease modeling and drug testing (Kolesky et al., 2014; Horvath et al., 2015; Lee A. et al., 2019; Lee H. et al., 2019; Theodoridis et al., 2019; Daly et al., 2020). However, there are still challenges and limitations to be addressed before this novel approach is fully adopted by researchers. For example, not all biomaterials are compatible with AD fabrication methods for the recapitulation of some complex micro- and nano-features, while printing modules and parameters, such as print speed, print pressure, and temperature as well as cell density in the bioink, can influence the cell-material dynamics during the printing process (Zhang and Wang, 2019). Nevertheless, as new methods for modulating biomaterial properties (e.g., new cross-linking mechanisms), along with advances in printing technologies—both software and hardware—are developed, we expect to see advances in the convergence of AD manufacturing and *in vitro* TE with the potential to leverage physicochemical cues and hence facilitate the development of more robust tissue equivalents.

Finally, a highly promising and popular method for building tissue equivalents, which has been favored by the emergence of the aforementioned AD manufacturing technology, is modular tissue engineering (Ouyang et al., 2020). Contrary to the top-down approach of the traditional TE paradigm, this bottom-up approach is based on fabricating living building blocks using cells (optionally together with biomolecules and/or biomaterials) which are then assembled to create more biomimetic customized tissue models (Nichol and Khademhosseini, 2009; Ouyang et al., 2020). Various methods of building and assembling these modular tissue blocks are being explored, including 3DP/bioprinting (Graham et al., 2017; Liu T. et al., 2019; Subbiah et al., 2020), micropatterning (De Gregorio et al., 2018), microfabrication of cell-ladden hydrogels (Onoe et al., 2013; Jeon et al., 2019; Figure 4E) and scaffolds (de Rutte et al., 2019), and self-assembly (Kato-Negishi et al., 2013; see Ouyang et al., 2020, for an extended review). Among other advantages, bottom-up tissue engineering approaches have gained a lot of attention because they offer a new means to generate vascularized tissues via modular assembly of pre-formed vascularized tissue blocks, a major challenge for tissue engineering at present (Nichol and Khademhosseini, 2009; Marga et al., 2012; Ouyang et al., 2020). In some cases, the versatility of these living tissue blocks allows not only for the modular assembly of vascularized tissues and organs (Homan et al., 2019), but also for the assembly of other complex tissue types (Miller et al., 2012; Magnan et al., 2020). It would be interesting to see if this strategy for vascularization of tissues could be also applied to promote innervation of biofabricated tissues and organs (Das et al., 2020).

### Engineering Methods to Recapitulate the Physicochemical Properties of the Native Niche

As discussed above, progress in materials science and engineering has enabled the development of functional/smart (bio)materials and platforms for TE applications by aiding the reconstruction and control of an environment that mimics key features of the natural niche. In the *in vivo* situation, cells have the capacity to generate, sense, integrate, and respond to systemic and local mechano-chemical cues and through interactions with neighboring cells and the surrounding ECM, they collectively generate tissues/organs with impressive structure and functionality (Brassard and Lutolf, 2019). Therefore, besides the appropriate source of cells and the materials with the appropriate physicochemical properties and microstructure, the exposure to biochemical and biophysical cues is of paramount importance for engineering tissues *in vitro* (Caddeo et al., 2017; Bao et al., 2018; Chen et al., 2019). Biophysical cues include bulk properties (e.g., viscoelasticity, stiffness, and porosity), as well as surface properties (e.g., roughness, guidance cues, charge, and wetting characteristics), while biochemical cues, besides the chemical structure, and composition of the materials, also refer to the presence of gradients of nutrients, signaling molecules, or even reprogramming factors, such as mRNA (Ledo et al., 2020a). In the following section, we discuss recent advances in reconstructing several niche properties and key aspects of the natural biochemical and mechanical signals, known to influence fundamental cellular processes, as well as methods of delivering such stimulants, to simulate the chemical signaling and biological pathways of the native environment and thus promote physiological cell growth and differentiation within the engineered tissue models.

#### Chemical/Biochemical Stimuli

*In vivo*, cellular responses are influenced by various spatiotemporal biochemical signals (Caddeo et al., 2017; Park et al., 2019). Within tissues, concentration gradients for soluble components, nutrients, metabolites as well as oxygen and pH exist and are essential for exerting pressures that can stimulate or inhibit basic cellular processes (Przyborski, 2017). The proximity of vasculature and blood vessels, the diffusion of molecules through the surrounding ECM and the metabolic activity of the organ/tissue, which regulate oxygen tension, nutrient consumption and cellular waste secretion and removal, affect these natural gradients and in turn the maintenance of physiological levels of chemotaxis and homeostasis (Langhans, 2018).

Since most tissue-engineered constructs lack a vascular network, cells rely for their survival on diffusion of nutrients and oxygen through the construct (Rouwkema et al., 2009). Along with cell culture media, the engineered (bio-)materials and ECM components of the 3D tissue equivalent can act as a reservoir of such molecules, as well as for soluble components that can enhance, stimulate or inhibit specific cellular functions and guide cells toward the desired output (Caddeo et al., 2017; Afewerki et al., 2019). Essentially, cell culture media is a cocktail of molecules and compounds that range from basic nutrients necessary for cell growth to biochemical stimulants with more specialized role, depending on the needs of the cell line(s) in culture (Table 2). For example, glucose is widely used as the main source of energy for cell metabolism, while serum (e.g., fetal bovine serum) forms the basis of culture media as the cocktail of hormones, growth factors and protease inhibitors, supplemented with buffering systems, inorganic salts, amino acids and proteins/peptides that promote cell growth and viability, while also control pH and osmolality of the cell culture environment (Yang and Xiong, 2012; Carter and Shieh, 2015b; Salazar et al., 2016). A type of biomolecule in media formulations with more specialized role is growth factors (GFs). GFs are used as culture media additives due to their key role in multiple signaling pathways between cells and their environment as well as in fundamental cellular processes. For instance, bone morphogenetic proteins (BMPs) stimulate bone cell differentiation, vascular endothelial growth factors (VEGFs) stimulate blood vessel differentiation (angiogenesis), while GFs, such as epidermal growth factor (EGF), regulate a wide variety of functions in both epithelial and mesenchymal cells (De et al., 2013; Yao and Asayama, 2017). In addition to providing a biochemical link for enhancing cell communication (Gonçalves et al., 2013), addition of GFs in the cellular microenvironment is necessary for the reconstruction of the native tissue niche, where they are part of an extensive cross-talk between cell membrane receptors and ECM components (Brizzi et al., 2012). This is particularly important in the case of stem cells as well as for the derivation and maintenance of various types of organoids (Urbischek et al., 2019) the self-organization and maturation processes of which require a spatially homogeneous cocktail of specific growth factors (e.g., R-spondins and Noggin) and other signaling molecules (Brassard and Lutolf, 2019).

**Table 2:** Common biomolecules in conventional cell culture media.

**Types of biomolecules**	**Role**	**Example**
Carbohydrates	Source of energy	Glucose Galactose
Amino acids	Protein synthesis, Secondary source of energy, Regulation of cell proliferation and density, Stimulation of growth and enhancement of cell viability	L-glutamine L-cysteine L-Lysine
Proteins and peptides	Binding of water, salts, free fatty acids, hormones, and vitamins Removal of toxic substances, Protection against proteolysis, Promotion of cell attachment	Albumin Transferrin Fibronectin Aprotinin
Growth factors	Cell signaling and communication, Mediation of processes such as proliferation, differentiation, wound healing, and tissue maturation	Bone morphogenetic proteins (BMPs) Vascular endothelial growth factors (VEGFs) Epidermal growth factor (EGF)
Cytokines	Cell Signaling and communication Stimulation of cells toward differentiation pathways Modeling hematoimmune response of tissues	Interleukins (IL) Tumor necrosis factor a (TNF-a)
Vitamins	Cell growth and proliferation Enzyme co-factors	Vitamin B group

Another class of biomolecules used to engineer the *in vitro* niche and mimic native signaling networks is cytokines. Apart from their modulatory role in the hematoimmune system, cytokines produced by a broad range of cells (e.g., lymphocytes, endothelial cells, and fibroblasts),—depending on the type and state of cell—, have also been found immobilized in the ECM, forming a complex functional network within the body, exerting systemic effects that go beyond their immunomodulatory role (Morán et al., 2013). Hence, they are now being employed to engineer the microenvironment of tissue equivalents, not only as agents that enrich the cell culture media, but also as components of the (bio)material blends used to support the 3D culture system. For example, in a perfusion-based bioreactor model of human bone marrow, addition of hematopoietic cytokines (i.e., thrombopoietin, stem cell factor, and Fms-related tyrosine kinase 3 ligand) significantly aided the establishment of a xeno-free environment that in turn favored the expansion of hematopoietic stem cells (Bourgine et al., 2018), while controlled release of BMP and VEGF blended in bone-mimetic substrates was shown to exert a synergistic effect on stimulation of osteoblasts (Bao et al., 2017). Finally, it is worth noting the efforts on substituting animal derived-sera with human (Muraglia et al., 2017; Heger et al., 2018) or synthetic serum (Ejiri et al., 2015; Patel et al., 2015), as these have been shown to better support cell growth and behavior in 3D without compromising the results (Heger et al., 2018). In addition, standardization of such sera formulations will facilitate the development of completely animal-free cell systems and tissue equivalents, better capturing the native biochemical environment of specific cell types (Ejiri et al., 2015).

#### Physical/Biophysical Stimuli

Besides biochemical cues, each cell within the native tissu e is subject to a unique mechanical environment defined by gradients of intracellular and extracellular forces, the interactions with neighboring cells and the surrounding ECM (Caddeo et al., 2017; Brassard and Lutolf, 2019). Via mechanotransduction mechanisms, cells respond to these biophysical stresses and transduce the mechanical stimuli into biochemical signals, modifying their behavior (e.g., proliferation rate, shape, and migration). In addition, cells rearrange their cytoskeleton and cell membrane positioning and produce and exert endogenous contractile forces in the surrounding microenvironment, remodeling the ECM. This dynamic reciprocity of biophysical cues is constantly reshaping cells and the native niche structure and is associated with different cellular functions and tissue homeostasis (Xu et al., 2009; Humphrey et al., 2014; Kratochvil et al., 2019). Along with the physical properties and microarchitectural features of the tissue engineering materials, it has now been established that experimental platforms of tissue equivalents should acknowledge and incorporate the physiological biophysical variables to successfully imitate *in vitro* the dynamic interplay between cells and their exterior (Humphrey et al., 2014; Przyborski, 2017).

Over the last couple of decades, efforts are focusing on better understanding the effects of mechanical stimuli on cells and on addressing the challenges of reconstituting biophysical cues of physiological and diseased conditions *in vitro*. Of particular interest is the application of fluid shear stress that several tissues and cells within the body experience (Delon et al., 2019). For example, blood flow and pressure exert on endothelial cells one of the greatest forces within mammalian organisms (1–5 Pa) (Baeyens et al., 2016). In response to these forces, endothelial cells alter their morphology and orientation, which in turn regulates vessel physiology, function, and remodeling activity accordingly. In addition, endothelial cells transduce the frictional blood flow force into biochemical signals via specialized mechanisms that shape the ability of the vascular system to effectively perfuse all tissues. Alterations in the nature of these forces or in the mechanotransduction mechanism have been shown to contribute to major vasculature diseases (Kamiya and Ando, 1996; Kadohama et al., 2007; Baeyens et al., 2016; Chistiakov et al., 2017). Epithelial cells also experience fluid shear stress (e.g., peristalsis in the intestine), which affects both their structure and function. Several *in vitro* studies have revealed the importance of this mechanical cue in the formation of microvilli in the apical surface of various epithelial cell types, including intestinal (Delon et al., 2019), lung (Stucki et al., 2018), and placental (Miura et al., 2015), highlighting the importance of incorporating such biophysical cues in bioengineering applications.

The effects of biophysical cues in advancing the relevance of *in vitro* cellular and tissue models has also been explored in the context of stimulating cells to enhance their functional and phenotypical characteristics or to trigger the differentiation of stem cells toward the desired lineage. Mechanical stretching has long been an attractive experimental strategy for controlling cell growth, gene expression, lineage commitment, and differentiation and thus successfully engineering mechanically functional tissues, such as cardiac, lung, vasculature, and bone (Diederichs et al., 2010; Riehl et al., 2012). For example, cyclic stretch was shown to enhance the viability and functional maturation of 3D cardiac tissue constructs based on human embryonic stem cell-derived cardiomyocytes seeded on gelatin-based scaffolds (Mihic et al., 2014). In another study, Fang, et al. highlight the potential of mechanical stretch for enhancing stem cell behavior and regulating their fate. By applying cyclic stretch to human adipose-derived stem cells (hADSCs), the authors found that stretching significantly promoted the proliferation, adhesion, and migration of hADSCs, it suppressed apoptosis and adipogenesis, while it enhanced osteogenesis (Fang et al., 2019).

Besides mechanical cues, biophysical cues also involve electrical or magnetic fields, ultrasound stimulation and photostimulation (Ding et al., 2017; Chen et al., 2019). Due to the strong presence of bioelectricity (e.g., cell membrane potential, trans-epithelial potential found in all types of epithelial tissues) and its effects on *in vivo* systems, electrical stimulation has drawn a lot of attention for its potential benefits in tissue engineering (Balint et al., 2013; see McCaig et al., 2005, for an extended review on bioelectricity). Several *in vitro* studies have revealed the effects of electrical stimulation on various biological events both on cellular and tissue level, spanning from improved cellular migration and differentiation to enhanced wound healing and nerve regeneration (Vodovnik et al., 1992; Llucià-Valldeperas et al., 2015; Snyder et al., 2017; Srirussamee et al., 2019). In the recent years, these effects have also been explored in 3D culture setups. For example, Kumar et al., studied the effect of external dynamic electric field as a guiding cue for osteoblasts seeded on 3D printed porous titanium alloy scaffolds. Their findings suggest that the presence of electric field, under dynamic conditions, had a positive effect on proliferation, growth, and expression level of prominent adhesion and cytoskeletal proteins, as well as on cell-cell interactions (Kumar et al., 2016; Iandolo et al., 2020). However, the emergence of electroactive polymers as a new class of smart materials has brought to the fore the potential of combining materials suitable for TE with electrical stimulation. Early work on 2D cell culture assays based on polypyrrole, for example, has shown that such materials can support the growth, proliferation, and differentiation of mammalian cells (Zelikin et al., 2002) as well as stimulation of neurite outgrowth (Schmidt et al., 1997) or enhancement of osteogenic commitment of bone marrow stromal cells (Shastri et al., 1999) upon application of electrical fields. Moreover, carbon nanotubes (CNTs) have been shown to promote cardiomyocyte maturation (Martinelli et al., 2012) as well as to enhance the performance of engineered neurons and neural networks (Cellot et al., 2009; Fabbro et al., 2012), among other applications. Nowadays, it is well established that such materials can act both as substrates for cell attachment and tissue growth and as bioactive elements for regulating cellular activities within 3D tissue culture systems (Balint et al., 2013; Chen et al., 2019). The use of conducting materials in regulating stem cell function through electrical stimulation in 3D microenvironments was exemplified recently by co-culturing human adipose-derived MSCs (hADMSCs) and umbilical vein endothelial cells (HUVECs) in an electrically conducting polypyrrole/chitosan scaffold, demonstrating enhanced autocrine signaling, promoting the cellular functions of the co-culture system (Zhang et al., 2018). In another study, Zhu et al. developed carbon nanofibrous scaffolds with enhanced electrical conductivity and mechanical flexibility and demonstrated that sufficient support of stem cell-derived neuron-like cells, while application of biphasic electrical stimulation enhanced differentiation and maturation of these cells, as evidenced by the upregulation of the relevant neuronal biomarkers (Zhu et al., 2018). Finally, earlier this year, Iandolo et al. developed highly porous electroactive PEDOT:PSS and collagen type I composite scaffolds that supported neural crest-derived stem cell (NCSC) culture and osteogenic differentiation, without the need for scaffold pre-conditioning. The modulation of mechanical and electrical properties induced by collagen blending provided a new means for directing cell fate and response, as well as a tool for cell-based monitoring and stimulation applications (Iandolo et al., 2020).

#### Spatiotemporal Delivery and Control of Biochemical and Biophysical Cues in Engineered Tissue Equivalents

As discussed in previous sections, advancements in fabrication technologies and material engineering have enabled the development of tissue engineering substrates that can present cells with the necessary cues to finely elicit a plethora of cellular functions and signaling mechanisms (Leijten et al., 2017). However, our understanding of cell-material interactions so far has been based mainly on static culture systems, while the *in vivo* array of biochemical and biophysical signals changes over space and time. Even though mimicking natural gradients in 3D culture platforms is possible, the thickness of the culture construct, as well as the competition of cells, can limit the diffusion of nutrients, oxygen, growth factors, and other signaling molecules and due to inhomogeneous distribution, cells located in the middle of the construct might not have access to sufficient supply of those molecules and thus behave differently from cells that are closer to the engineered tissue surface (Levorson et al., 2011; Caddeo et al., 2017). In turn, this results in non-uniform cell proliferation and matrix deposition and in inhomogeneous tissue formation (Gholipourmalekabadi et al., 2016). Therefore, a lot of focus has now shifted to developing culture platforms that can dynamically recapitulate *in vitro* the native tissue spatio-temporal variation of signals. Such engineered platforms have the potential to facilitate better understanding and to provide more degrees of flexibility and control over cell function and fate and thus to eventually build tissues that better emulate the dynamics of the *in vivo* conditions (Leijten et al., 2017).

From the materials engineering point of view, even though numerous matrix-based techniques for delivering physicochemical cues, such as blending cell-adhesive ligands within scaffold materials (Gallagher et al., 2020) or micropatterning growth factors in hydrogels (Jeon et al., 2018), have been shown to improve cell behavior, this approach lacks the possibility to fine-tune and precisely control the timing of delivery, which is also important for cell survival and fate. In the case of MSCs, for instance, it has been shown that the RGD cell-adhesive motif is essential for stem cell survival at the early stages of 3D culture in PEG hydrogels, while removal of ligands at later stages does not compromise the viability of cells, but rather improves their differentiation (Kloxin et al., 2009). To overcome limitations in delivering natural cues in a spatiotemporal manner the development of dynamic biomaterials that allow for reversible modulation of the physicochemical properties and on-demand release of the desired molecules via either cell-mediated or user-mediated mechanisms, represents a promising strategy (Willerth et al., 2008; Leijten et al., 2017; Schneeberger et al., 2017; Cimmino et al., 2018; Kratochvil et al., 2019; Xu et al., 2020). A characteristic example where the delivery of physicochemical cues is mediated by cell activity is remodelable 3D hydrogels. Madl et al. showed recently that in elastin-like protein hydrogels, prior to chemically-induced differentiation, a critical amount of matrix remodeling is necessary to maintain the stemness and to enhance the differentiation capacity of neural progenitor cells into astrocytes and mature neurotransmitter-responsive neurons, via a mechanism regulated by cadherin cell–cell contacts and catenin-mediated activation of Yes-Associated Protein (YAP) expression (Madl et al., 2019). These findings highlight the potential of bioresponsive materials as attractive tissue engineering platforms that enable both expansion and subsequent differentiation of stem cells toward the desired tissue within a single cell culture setup. On the other hand, materials can be engineered to respond to user-directed stimuli as a means to fine-tune the presentation of spatiotemporal cues to cells and thus to direct and modulate interactions within the biological system (Cimmino et al., 2018). An example of this approach is the use of protease-cleavable peptides in 3D materials, as shown in a recent publication by Guo et al. The researchers, combining bio-orthogonal click chemistry and protein engineering, developed PEG-based multi-layered hydrogels with spatially-defined regions of immobilized proteins, where exogenous application of enzymes for triggering the temporal release and removal of select proteins was shown to be a promising tool for controlling cellular microenvironments (Guo et al., 2017). Due to their spatiotemporal tunability, materials responsive to light, temperature or electrical fields have also been called for, to allow triggering of the release of ECM-presented cues from a 3D substrate to the cell culture microenvironment, thus directing advanced cellular fates within tissue engineering platforms (Wylie et al., 2011; Deforest and Tirrell, 2015; Cimmino et al., 2018; Ruskowitz and Deforest, 2018; Kratochvil et al., 2019; Shadish et al., 2019).

Sophisticated bioreactor systems can be used to fine-tune culture parameters and to reproduce an *in vitro* tissue-specific physiological microenvironment capable of overcoming diffusion limits and oxygenation for better control or even coupled delivery of chemical and mechanical cues (Levorson et al., 2011; Hansmann et al., 2013; Schmid et al., 2018). For example, a bioreactor system developed by Zohar et al. enabled the investigation of the direct flow-induced shear stress on vascularization of poly(L-lactic acid)/poly(lactic-co-glycolic acid) (PLLA/PLGA) scaffolds and showed that flow conditions enhance vascular network formation and maturation (Zohar et al., 2018). Charoensook et al. created a bioreactor-based functional *in vitro* model of the neuromuscular junction by cultivation of transdifferentiated myocytes and stem cell-derived motoneurons, where electrical stimulation resulted in improved maturation and function of motoneurons and myocytes, as well as exhibiting physiological response to drugs, thus suggesting its potential as a pharmacological screening platform and controlled studies of neuromuscular diseases (Charoensook et al., 2017). Furthermore, an all-in-one bioreactor approach facilitated the reconstruction and control of a more physiologically relevant 3D cardiac tissue microenvironment by combining, within a single chamber, electrical stimulation of the cardiac tissue, bidirectional interstitial fluid flow and on-line monitoring, and analysis of tissue functionality during culture (Visone et al., 2018).

One of the most promising technologies for bridging the gap between *in vitro* and *in vivo* systems is organs-on-chips (OOCs), alternatively called microphysiological *in vitro* models. OOCs technology has emerged from the combination of recent advances in microengineering and fluidic physics with trends in growing cells in 3D, allowing for the development of models that more faithfully recapitulate key features of specific human tissues and their interactions (Ramadan and Zourob, 2020). The design of the vast majority of OOC models are based on (micro-)fluidic devices, fabricated by soft-lithographic techniques, with continuously perfused chambers inhabited by living cells arranged in a biomimetic manner, while facilitating precise control over delivery of nutrients and spatiotemporal tuning of oxygen and pH gradients (Bein et al., 2018; Ronaldson-Bouchard and Vunjak-Novakovic, 2018). Among the added benefits of using such systems are the continuous supply of nutrients and removal of waste, the unparalleled, independent control over multiple key factors of the cell system, the possibility for *in situ*, high precision and automated monitoring and sample analysis, as well as the ability to interface different cellular compartments for enhanced cell cross-talk and exchange of signaling molecules and growth factors. But, what creates an enormous potential for enhancing the physiological relevance of the *in vitro* cell systems, spurring new, unforeseeable applications of this technology, is the combination of a biomimetic niche with accurate, precise, and coupled delivery of more complex biochemical and biophysical cues (Bein et al., 2018; Ramadan and Zourob, 2020). Even though most of the attention OOCs have gained is focused on pharmacology and pre-clinical drug screening applications, as low-cost and animal-free alternative tool (Ramadan and Zourob, 2020), it is clear that the principle behind this technology lines perfectly with the TE paradigm and scope: convergence of cells with the advanced chip technology-biomaterials and delivery of physiologically relevant cues toward more robust tissue equivalents. As a result, various research groups around the world, both in the academic and industrial sectors, have developed a broad range of OOCs, mimicking the human gut (Ramadan and Jing, 2016; Kasendra et al., 2018; Shin et al., 2019), liver (Delalat et al., 2018; Jang et al., 2019), kidney (Jang et al., 2013; Chang et al., 2017; Yin et al., 2020), lung (Huh et al., 2012; Stucki et al., 2018; Felder et al., 2019), blood-brain barrier (Kilic et al., 2016; Wevers et al., 2018), bone (Marturano-Kruik et al., 2018), and vasculature (Schimek et al., 2013; Jeon et al., 2015) in both healthy and pathophysiological conditions, such as infection (Villenave et al., 2017; Ortega-Prieto et al., 2018) and cancer (Ayuso et al., 2016; Hassell et al., 2017; Hao et al., 2018; Carvalho et al., 2019), as well as for the interaction of multiple organs, as first showcased by Shuler et al. and more recently by others, toward multi-organ and whole-body microsystems (Miller and Shuler, 2016; Vernetti et al., 2017; Edington et al., 2018; Herland et al., 2020), to study collective responses to drugs or disease inducing agents and inter-organ communication.

## Applications of 3D Biomimetic Cultures and Tissue Equivalents

### Modeling Human Health and Disease

The transformation of the field toward more *in-vivo-*like human models is still ongoing, facilitated by the technological advancements in 3D cell platforms. Their deployment in early biomedical applications has already given prominence to the usefulness and validity of such models, leading to a paradigm shift in our understanding of human cell and tissue biology (Chen, 2016; Przyborski, 2017). Several studies over the past decades have showcased the powerful potential of 3D *in vitro* models in addressing questions specific to the human biology that are challenging, if not impossible, to answer with animal models and conventional biological assays. Additionally, increasing evidence suggests that such models are highly valuable for modeling pathophysiological conditions to study the disease onset and progression mechanisms, to identify pathogenic factors and potential therapeutics in the pre-clinical and clinical level (Chen, 2016).

**Figure 5:**
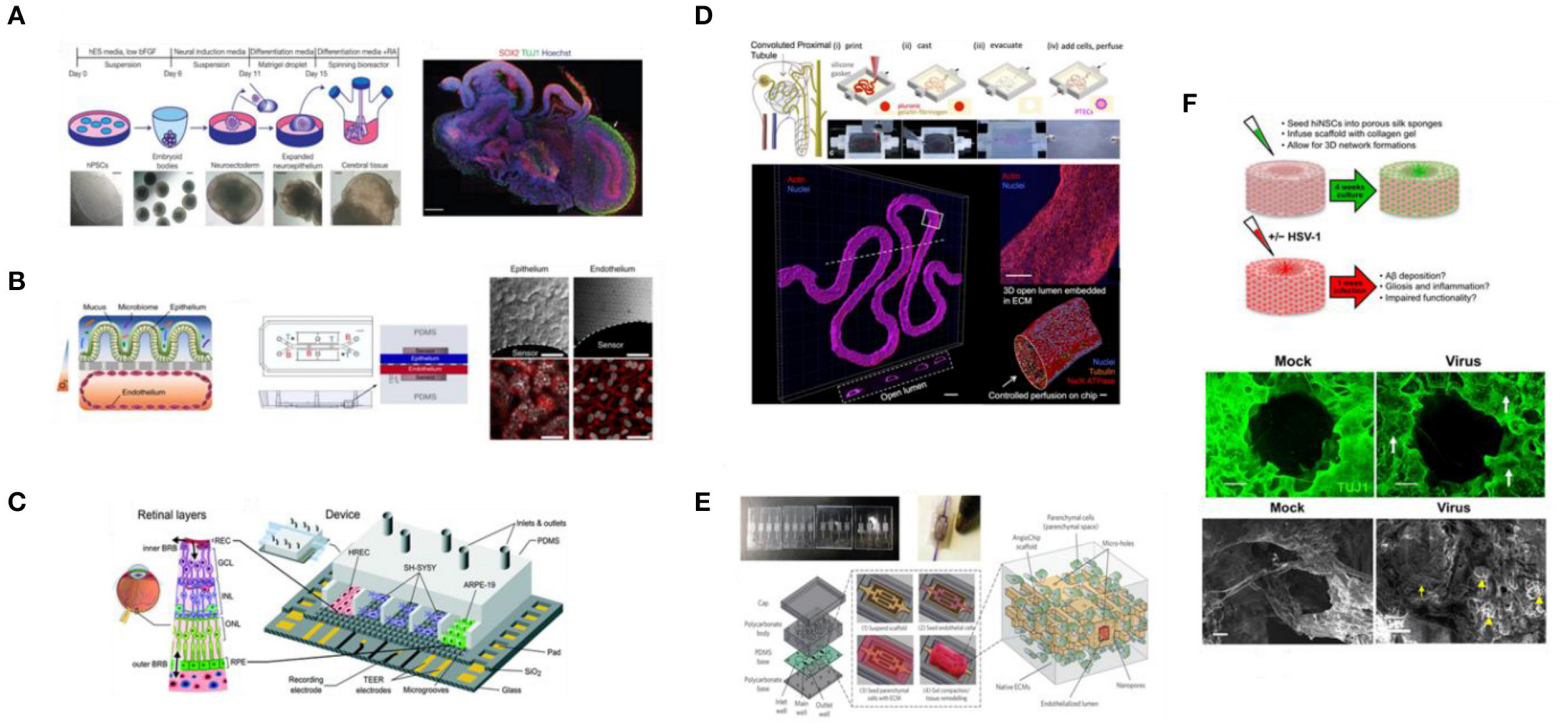
Biomimetic models of human (patho-)physiology. **(A)** Schematic representation of the cerebral organoid generation method (left) and immunohisto- chemistry image of a cerebral organoid section, revealing its complex morphology with regions containing neural progenitors (SOX2, red) and neurons (TUJ1, green) (right). Adapted from Lancaster et al. (2013). **(B)** Schematic illustration of the two-channel microfluidic Organ Chip device with an oxygen gradient (color scale) (left), schematic representation of the Intestine Chip with its embedded oxygen sensing unit (middle), and microscopy images of the Intestine Chip showing the morphology of the intestinal epithelium and endothelium morphology in the respective channels (right). Adapted from Jalili-Firoozinezhad et al. (2019). **(C)** Schematic illustration of a microfluidic chip culture device for modeling the blood-retinal barrier, with integrated TEER measurement electrodes. Reproduced from Yeste et al. (2018). **(D)** Schematic of the nephron convoluted proximal tubule and illustration of the bioprinting process steps, with corresponding images, in the fabrication of 3D convoluted perfusable proximal tubules (top), confocal microscopy 3D renderings of the bioprinted convoluted renal proximal tubule (bottom). Reproduced from Homan et al. (2016) under the Creative Commons CC BY license. **(E)** Images of the AngioChip scaffold (top) and schematics of the assembly of the bioreactor and of the vascularized tissue (bottom and right). Reproduced from Zhang et al. (2016a). **(F)** illustration of a 3D human brain-like model for modeling HSV infection (top), Images of the brain model showing b-III tubulin (TUJI1), and beta amyloid (Ab) immunostaining (middle), and scanning electron micrographs revealing the effects of HSV-1 infection on the brain-like tissue constructs (bottom). Adapted from Cairns et al. (2020) under the Creative Commons Attribution-Non Commercial license.

As discussed earlier, the advent of human organoids as systems strikingly similar to the *in vivo* environment has provided researchers with the unique opportunity to recreate the human physiology and architecture *in vitro* (Kim et al., 2020). A study that laid the foundation for the use of organoids for biomedical applications, was the generation of cerebral organoids in 2013. Lancaster and Knoblich used hiPSCs to establish an organoid culture system with various discrete, independent brain regions, including the cerebral cortex with progenitor populations, that give rise to mature and functional cortical neuron subtypes, recapitulating the formation of neural tissue in human brain development stages. In addition to this healthy brain tissue, they used patient-specific iPSCs and developed cerebral organoids that bore clear characteristics of the microcephaly neurodevelopmental disorder. This system helped them to address questions about the disease in a way that would not have been possible by growing neurons in flat surfaces or by engineering mouse models, due to lack of the necessary niche and interspecies differences, respectively (Lancaster et al., 2013; Figure 5A). Since then, organoids have played a prominent role in enhancing our understanding of various biological phenomena, including development and organogenesis (Karzbrun et al., 2018; Trisno et al., 2018; Vyas et al., 2018; Rossi et al., 2019; Shi et al., 2020), infectious biology (Forbester et al., 2015; Leslie et al., 2015; Karve et al., 2017; Heo et al., 2018; Lamers et al., 2020), cancer (Li et al., 2018; Nagle et al., 2018; Fusco et al., 2019; Ooft et al., 2019), and other diseases. Among others, the recent study by Sachs et al. on long-term expanding lung organoids clearly shows the versatility of these experimental tissue formats in faithfully recapitulating the adult epithelial airway structure and function from both healthy individuals and from patients with cystic fibrosis, lung cancer, and viral infections (Sachs et al., 2019). Finally, the valuable contribution of organoids in disease modeling is also showcased by the great efforts to build novel biomedical resources where samples from minimal amounts of tissue biopsies are used to generate patient-derived organoids, stored for future research, known as living biobanks (Sachs et al., 2018; Yan et al., 2018; Kim et al., 2020; Nelson et al., 2020; Simpson et al., 2020). The potential of such biomedical resources for modeling disease and investigating therapeutic strategies, even at the personalized level, is nicely exemplified in a study published earlier this year that presents methods for generating and biobanking patient-derived glioblastoma organoids (GBOs) that recapitulate and preserve the cellular and mutational diversity of the corresponding glioblastomas, reflecting inter- and intra-tumoral heterogeneity, holding great promise as a precision medicine tool for diagnosis and treatment (Jacob et al., 2020).

In parallel, the growing body of literature over the past years reflects the tremendous effort and progress in bioengineering tissue equivalents that allow for interfacing with vasculature and enable more in-depth investigation of tissue structure, homeostasis and pathophysiology, as well as communication between tissues and their surroundings. As discussed in the previous sections, successful engineering of tissues *in vitro* requires careful consideration of the cell sources, the type of material and the fabrication method as well as of the integration of the relevant biophysical and biochemical cues, all of which highly depend on the native tissue structure and function and on the biological question to be addressed with the model. For example, the delineation of tissue compartments and the regulation of the passage of ions and solutes by epithelia and endothelia are of particular importance for the recreation of such barrier tissues. While early strategies, relied on permeable supports to separate the apical and basal compartments of the barrier-forming monolayers (Lea, 2015; Pearce et al., 2018), microphysiological systems and OOC approaches are taking this concept a step further, by exploiting novel bioengineering techniques to create compartmentalized barrier models with a more biomimetic interface between endothelial and epithelial tissues, combined with delivery of essential environmental cues (Bein et al., 2018). The work of Huh et al. was among the first attempts to this end. The researchers created a “lung-on-a-chip” that models human lung function in both normal and disease states, by co-culturing microvascular endothelial cells (i.e., bottom, microvascular compartment) and alveolar epithelial cells (i.e., top, alveolar compartment) in parallel microchannels separated by a thin semipermeable membrane, that also enabled the establishment of an air-liquid interface environment, where cell culture medium was perfused via the microvascular channels while the alveolar channel was filled with air. Cyclic stretch of the tissue via application of vacuum to the side compartments of the parallel channels was shown to successfully mimic the breathing motion of the lung, while concurrent administration of the cytokine interleukin-2 (IL-2) to the microvascular channel was shown to compromise the barrier, reproducing the pulmonary leakage, which is a characteristic symptom of the pulmonary oedema (Huh et al., 2012). Versions of human gut-on-a-chip were also developed with the same device, where the side vacuum chambers and the application of fluid flow were exploited to recreate the peristaltic motions, but were also shown to induce spontaneous formation of the characteristic intestinal villus-crypts structures (Kim and Ingber, 2013). More recently though, this system was modified to accommodate the development of a co-culture of mucus-producing human primary intestinal epithelium in the same compartment with stable communities of the human gut microbiota by applying a hypoxia gradient across the endothelium-epithelium interface, while simultaneously monitoring oxygen levels and intestinal barrier function (Jalili-Firoozinezhad et al., 2019; Figure 5B). In a different approach, Trietsch et al. developed their intestine-on-a-chip model in a membrane-free manner, where a functional intestinal barrier is formed in a lateral channel of a microfluidic chip, at the interface with an ECM gel that supports the basal side of the epithelium and facilitates access to the perfusion channel on the opposite side of the intestinal tubules (Trietsch et al., 2017). Another way to achieve compartmentalization and tissue-tissue interfaces within OOC systems, bypassing the use of membranes, was proposed by Yeste et al. who developed a microfluidic device where cells grow in parallel compartments that are highly interconnected via a grid of microgrooves, facilitating heterotypic cell-cell contact, and paracrine signaling, while integration of electrodes allows for in-line monitoring of the cell barrier integrity in each compartment. The device successfully supported the generation, maintenance and monitoring of a blood-retinal-barrier model, based on the co-culture of primary retinal endothelial cells (HREC), a human neuroblastoma cell line (SH-SY5Y), and a human retinal pigment epithelial cell line (ARPE-19) (Figure 5C), highlighting the necessity of compartmentalization in enhancing the robustness of barrier models as well as the added benefits of integrating in-line monitoring units (Yeste et al., 2018). OOC technology has also significantly contributed in dissecting *in vitro* the mechanisms behind various (patho-)physiological mechanisms of the brain (Haring et al., 2017). Herland et al., for instance, were able to study human neurovascular function and inflammation in a 3D model of the human blood-brain barrier (BBB) within a microfluidic chip and to reveal the distinct contributions of astrocytes and pericytes to neuroinflammation (Herland et al., 2016). Kilic et al. proposed a brain-on-chip model suitable for studying the migration of human neural progenitors in response to chemotactic cues (Kilic et al., 2016), while Park et al., proposed a microfluidic device for the brain-like interstitial perfusion of neurospheroids and tested the toxicity of amyloid-β, showcasing the validity of brain-on-chips in modeling and studying neurodegenerative diseases as well (Park et al., 2015).

Although OOC models have been shown to better capture the *in vivo* situation, compared to conventional 2D culture formats, often such models are quasi-3D, forming an intermediate stage between 2D and 3D cell culture microenvironments rather than truly biomimetic tissues. To overcome this limitation, OOC technologies are now going beyond 2D, utilizing gels and scaffolds as tissue growth templates, toward generating 3D tissue equivalents of high biomimicry (Terrell et al., 2020), as shown recently in reports on the fabrication of 3D convoluted, luminal tissue architectures (Massa et al., 2017; Manousiouthakis et al., 2019; Wang X. et al., 2020). For instance, 3D human renal proximal tubules were engineered via combined bioprinting with 3D cell culture and OOC principles. The tissue construct was housed in perfusable chips and embedded within an extracellular matrix that supported the active perfusion, growth, differentiation, and maintenance of the proximal tubule epithelium for over two months, during which the nephron-like tissue exhibited significantly enhanced epithelial morphology and functional properties (e.g., brush border, basement membrane protein deposition, basolateral interdigitations, enhanced cell height, megalin expression, and albumin uptake), as well as *in-vivo-*like response upon delivery of nephrotoxin and cyclosporine A (Homan et al., 2016; Figure 5D). Robust 3D vascular models that more faithfully capture the natural milieu have also been called for interfacing with different tissue equivalents (Morgan et al., 2013; Kolesky et al., 2016). In an attempt to overcome the challenges in the choice of material for vasculature engineering as well as to address the challenge of co-cultivating parenchymal cells with vasculature in 3D, Zhang et al. created the AngioChip. This is a stable biodegradable scaffold that consists of a perfusable 3D, branched, luminal microchannel network with thin, flexible but yet mechanically compliant walls, lined by endothelial cells, and surrounded by a tunable matrix that supports the assembly of parenchymal cells. In addition, the walls feature nano-pores and micro-holes that were shown to enhance permeability, to facilitate efficient molecular exchange, intercellular crosstalk (Figure 5E), as well as extravasation of monocytes and endothelial cells upon biomolecular stimulation, as showcased via the successful vascularization of hepatic and cardiac tissues. The precise placement of endothelial and parenchymal cells, in a simple to operate format, the control of the initial architecture of the vasculature as well as the potential to fine-tune the vessel permeability to match the requirements of different organ models, highlight the AngioChip as a versatile tool for cultivating the vasculature in tissue engineering platforms (Zhang et al., 2016a). Progress in modeling the human brain has also been made with the emergence of *in vitro* TE technology. Researchers have long studied the multi-layered, hierarchical brain architecture and complex physiology, but recapitulating the entire brain *in vitro* still remains a huge challenge, in part because of this complexity but also due to the complications associated with the available technology (Lozano et al., 2015). Novel cell sources combined with new materials and technological platforms have yielded new tools for building functional brain-like tissues, pushing further the borders of our understanding of the human brain. This is also particularly important for neurodevelopmental and neurodegenerative studies where translation of findings to the clinic is hindered by interspecies differences (e.g., cognition), among other factors (Hackam and Redelmeier, 2006; Hartung, 2013). For instance, in a pioneering study Tang-Schomer et al. developed a 3D brain-like cortical tissue construct using primary cortical neurons in a silk fibroin/collagen gel composite scaffold as a support for the 3D axon connections, that was able to reproduce the compartmentalization of gray and white matter as well as the *in vivo* relevant biochemical and electrophysiological outcomes necessary for the assessment of both brain physiology and brain disorders (Tang-Schomer et al., 2014). Optimization of this silk-fibroin scaffolds, along with the development of a technique to generate expandable and rapidly differentiating human-induced neural stem cell (hiNSC) lines, has enabled this group to further advance their approach in building 3D brain tissue equivalents that allow for long term studies of neural tissue in various conditions, such as neurodegeneration, brain tumors and injury (Chwalek et al., 2015; Cairns et al., 2016; Cantley et al., 2018; Sood et al., 2019; Rouleau et al., 2020). A remarkable application of this system though was reported earlier this year where the implication of herpes simplex virus type I (HSV-1) as a causative agent of Alzheimer's Disease was investigated. The 3D bioengineered brain model was able to reproduce the herpes-induced AD pathophysiology, encompassing features of the *in vivo* physiological human disease, including Aβ plaque formation, neuronal loss, reactive gliosis, neuroinflammation, and diminished neural network functionality, reflecting the great progress and the tremendous potential of 3D TE approaches to address the critical need in building robust and physiologically relevant 3D human tissues (Cairns et al., 2020; Figure 5F).

### Drug and Treatment Development

**Figure 6:**
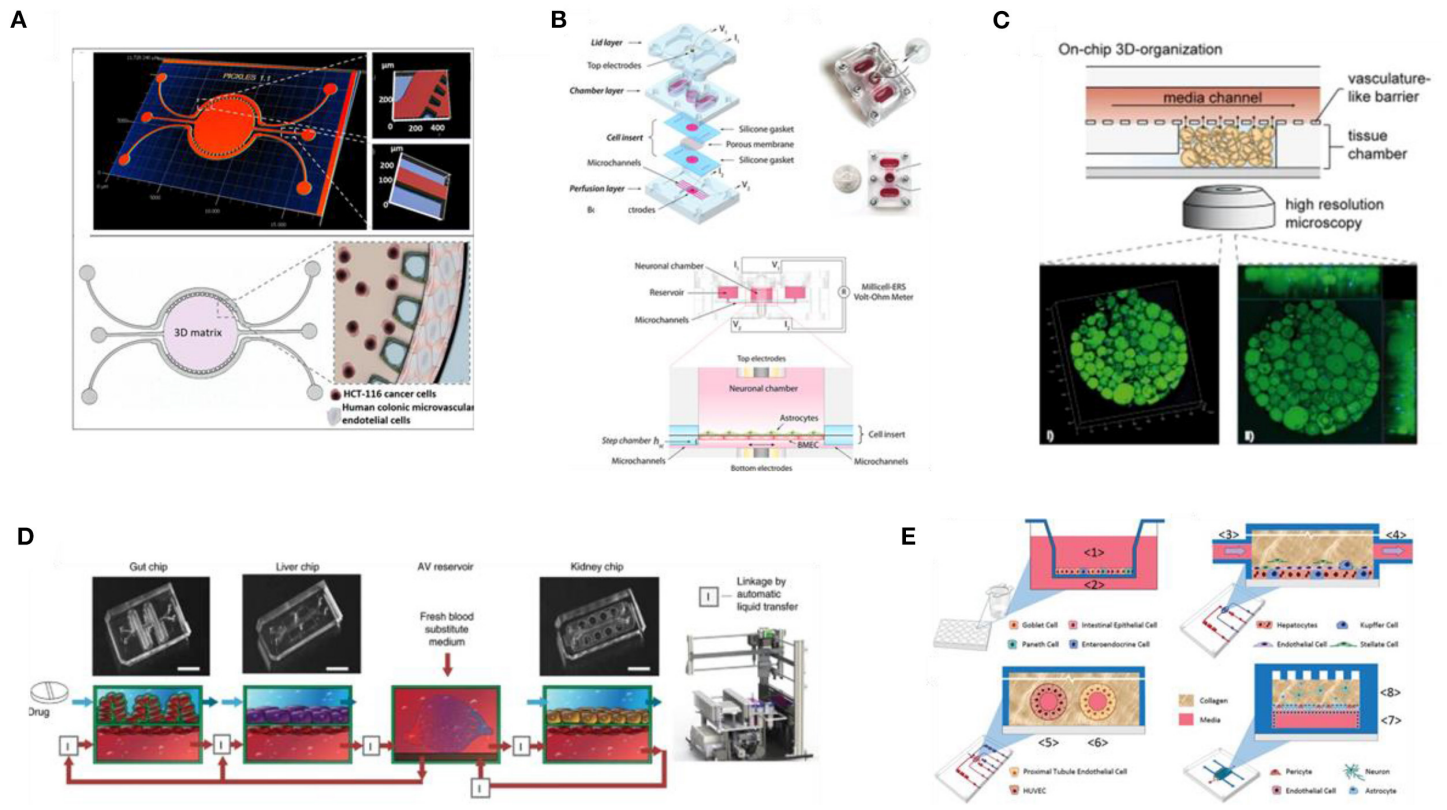
Novel *in vitro* human models for drug screening and testing applications. **(A)** 3D map image of the colorectal tumor-on-a-chip system features (top) and schematic of the chip design and culture setup (bottom). Adapted from Carvalho et al. (2019) under the Creative Commons Attribution-Non Commercial license. **(B)** Schematic illustration of a BBB-on-a-chip platform and assembly steps (top left), image of the assembled device (top right), cross-section of the assembled platform (middle), and zoom-in panel illustrating the device and cell culture setup (bottom). Reproduced from Wang et al. (2017). **(C)** Design of the human WAT-on-a-chip confocal image of the tissue construct generated in the device. Reproduced from Rogal et al. (2020) under the Creative Commons CC BY license. **(D)** Design and photographs of a first pass multi-organ chip system (top) and diagram of the fluidic coupling of the gut, liver, and kidney chips, containing vasculature compartments, and of the arteriovenous (AV) reservoir via an automated liquid handling instrument. Reproduced from Herland et al. (2020). **(E)** Schematic representation of the four organ systems used for the functional coupling of a human microphysiology system: intestine, liver, vascularized kidney, and BBB, including the neurovascular unit. Reproduced from Vernetti et al. (2017) under the Creative Commons Attribution International License.

3D biomimetic systems technology facilitates not only elucidation of disease biology and deeper understanding of the onset and progression mechanisms, but also identification and screening of potential drug candidates and therapies. Implementation of these systems in various stages of drug discovery and development is considered as a powerful alternative for addressing the challenges associated with the poor predictive power of existing preclinical models that, more often than not, is responsible for the high attrition rates of clinical trials (Roth and Singer, 2014; Caddeo et al., 2017; Ronaldson-Bouchard and Vunjak-Novakovic, 2018). 3D biomimetic cultures in the format of spheroids, organoids, hydrogels and scaffolds that more closely capture the disease physiology have already demonstrated the validity of this technology for drug development, screening, and toxicology assays (Sobrino et al., 2016; Wan et al., 2016; Villasante et al., 2017; Plummer et al., 2019; Han et al., 2020). However, OOCs in particular have gained a lot of attention for their potential to produce more predictive and accurate data by resolving the discrepancies in drug safety and efficacy observed between models currently used in preclinical and clinical stages of drug testing and hence accelerate the translational process (Haring et al., 2017; Low and Tagle, 2017; Ronaldson-Bouchard and Vunjak-Novakovic, 2018). The precise control of multicellular activities, the spatiotemporal distribution/delivery of relevant cues and the interface between different tissue types that OOCs offer has been exploited for the evaluation of novel anticancer therapies (Sontheimer-Phelps et al., 2019), among others (Ribas et al., 2016; Mittal et al., 2018). For example, Bai et al. demonstrated that a microfluidic platform, interfacing lung or bladder carcinoma aggregates with vessel-like structures, can serve as an *in vivo*-like surrogate for anti-invasive and anti-metastatic drug screening, revealing the role of signaling pathways involved in the drug action mechanism (Bai et al., 2015). More recently, a-tumor-on-a-chip platform, where the efficiency and toxicity of gemcitabine-loaded nanoparticles on Matrigel-embedded human colon cancer cells in contact with a 3D vessel-like colonic endothelium was successfully evaluated, was proposed as a precision onco-nanomedicine tool (Carvalho et al., 2019; Figure 6A). Despite the fact that oxygenation, a highly important parameter for the cancer microenvironment, is not taken into account in terms of monitoring and evaluation, both studies reflect the important developments made toward more accurate and precise screening of cancer therapeutics utilizing OOCs.

OOC platforms are also highly relevant for testing drug permeability and transport across the blood-brain-barrier (BBB). This distinctive tissue structure is made up of neurons, astrocytes, oligodendrocytes, microglia, smooth muscle cells, brain epithelial and endothelial cells, and pericytes embedded in the brain extra-cellular matrix (ECM), and bears the responsibility of maintaining brain homeostasis by supporting the neuronal activity and by tightly regulating the passage of metabolites, drugs, and other solutes from the peripheral blood into the central nervous system (Griep et al., 2013; Wang et al., 2017; Vatine et al., 2019). As OOC approaches allow for better compartmentalization and efficient assembly of the corresponding cellular components and environmental cues, thus more realistic reconstitution of the native tissue (Booth and Kim, 2012; Adriani et al., 2017; Wang et al., 2017; Campisi et al., 2018; Vatine et al., 2019), their utility in modeling various features of the BBB and their implementation in preclinical drug evaluation studies has gained a lot of attention recently, particularly for testing whether a drug designed to treat neurological diseases can actually cross the BBB to reach its target (Ronaldson-Bouchard and Vunjak-Novakovic, 2018). This was showcased by Wang et al. who proposed the fabrication of a BBB–on-a-chip with integrated TEER sensors for *in situ* monitoring of the barrier tissue integrity (Figure 6B). Even though the authors utilized primary astrocytes derived from rats, in a co-culture with human iPSC-derived brain microvascular endothelial cells (BMECs), their BBB microfluidic chip was able to generate drug permeability data comparable with *in vivo* values (Wang et al., 2017). In a different application, a compartmentalized, multilayer OOC device was shown to successfully maintain and monitor functional human white adipose tissue and fatty acid metabolism while also being applicable for testing tissue responsiveness to therapeutic compounds, useful for diabetes, and obesity studies (Rogal et al., 2020; Figure 6C).

Ultimately, what renders 3D biomimetic tissues and particularly OOCs even more attractive alternatives for drug and therapy development is their ability to be linked via their endothelium/vascular channel, in a way that mimics the drug distribution within the body, toward multi-organ systems for simulating pharmacokinetic and pharmacodynamic drug responses (Prantil-Baun et al., 2018; Ronaldson-Bouchard and Vunjak-Novakovic, 2018). This possibility has been recently explored by various studies, where different tissues were fluidically coupled to model the *in-vivo-*like organ interactions (Zhang et al., 2009; Maschmeyer et al., 2015a,b; Tsamandouras et al., 2017). A very recent characteristic example of such a multi-organ system was reported earlier this year, comprising organ-on-chip models of the gut, liver and kidney. The models were linked by their endothelium-lined channels through a robotic system circulating a common blood substitute that represents the systemic circulation, stored in a fluid-mixer reservoir that represents the arteriovenous (Figure 6D). The system was then used to model first-pass absorption, distribution, metabolism, excretion and toxicity of nicotine and cic-platin and quantitatively predict pharmacodynamics, and pharmacokinetic parameters, with results matching clinical data (Herland et al., 2020). In the same concept, Vernetti et al. coupled human MPS models representing the major absorption, metabolism and clearance organs (the jejunum, liver, and kidney) along with skeletal muscle and neurovascular models and evaluated the organ-specific processing, pharmacokinetic, and toxicity of terfenadine, trimethylamine (TMA)—as a potentially toxic microbiome metabolite—and vitamin D3. Their findings were consistent with clinical data, while they also discovered that trimethylamine-N-oxide (TMAO) can pass through the BBB (Vernetti et al., 2017; Figure 6E).

Without a doubt, this technology has the potential to transform drug discovery and development. However, there are still several challenges that should be addressed before the field realizes its tremendous potential. For example, with a few exceptions, most scaffold/hydrogel-based 3D models and OOCs primarily utilize cell lines, which in some cases may drift from the normal genotypic and phenotypic profile of the tissue of origin (Carter and Shieh, 2015a). Populating next-generation models with more relevant cells, such as iPSCs and organoid-derived patient specific cells can help overcome this limitation and better capture the tissue phenotype and thus to better mimic drug responses. This should be in line with the biological question the model is trying to address, recapitulating the specific features of the pathophysiology of interest. In addition, current OOC and MPS systems are fabricated predominantly with polydimethylsiloxane (PDMS), due to its ease-of-use, elasticity, optical transparency, and low-cost microfabrication. But issues related to the absorption of small hydrophobic molecules by this material severely compromise the validity of such systems in drug screening studies, pushing for transition to alternative, non-absorptive materials (Campbell et al., 2020). Also, 3D tissue equivalents and OOCs, as more sophisticated and multi-parametric models, require close control and synchronism of these different parameters to achieve the necessary functionality, particularly when long-term viability is the case (Sontheimer-Phelps et al., 2019). Moreover, in multi-organ systems, besides *in-vivo-*like sequential coupling of each counterpart, scaling must also be taken into account, to match flow volume and rate with cultured tissue mass in order to mimic the native conditions and to achieve the required level to support functionality (Wikswo et al., 2013; Bhatia and Ingber, 2014; Ronaldson-Bouchard and Vunjak-Novakovic, 2018; Ramadan and Zourob, 2020). Finally, for this technology to live up to its potential and to be successfully implemented in the drug development process, it is necessary to move from the proof-of-concept laboratory models toward more widely available prototypes to facilitate high-throughput screening and further validation that the models effectively mimic *in vivo* drug responses. Since OOCs is a multidisciplinary field, the overall progress of this technology and its implementation in drug development, among other applications, heavily relies on parallel advancements in the field of cell biology, materials, microengineering, and microfluidics. Toward this direction, the introduction of commercially available systems is already taking place, promoting the production of more automated, user-friendly OOCs, and will definitely help resolve current limitations (Zhang and Radisic, 2017). Nevertheless, to more efficiently employ this technology, more deep understanding of cell-material and cell-cell interactions and functions, as well as better understanding of the effects of biochemical and biophysical stimuli on the overall tissue structure and function is required.

### Sensing and Monitoring

As discussed during the previous sections, novel technologies for 3D TE *in vitro* offer unprecedented control over various parameters for the development and maintenance of the tissue equivalent of interest. In addition to fine-tuning the biological system parameters, this technology allows for the integration of in-line sensors that report not only on system parameters (e.g., flow rate, O2 levels, and pH), but also provide feedback on cellular activity, thus facilitating the study of a broad range of physiological phenomena (Bhatia and Ingber, 2014). Despite their inherent limitations, the majority of current approaches rely heavily on optical transducers, such as fluorescence microscopy, which is more a qualitative assay, that requires labeling (Kim S. et al., 2016; Sobrino et al., 2016), combined with downstream analysis of effluents to detect changes in gene expression and metabolite production (Curto et al., 2017; Roh et al., 2019). The use of invasive probes and the terminal nature of these assays, which sometimes requires harsh and lengthy protocols for sample preparation prior to imaging and analyzing, can be bypassed by advanced imaging techniques. For example, Raman spectroscopy has emerged as a suitable tool for non-invasive, *in situ* quality control of cells and substrates, as well as for real-time monitoring of physiologically relevant metabolites (Pudlas et al., 2011; Zbinden et al., 2020). Two-photon excitation microscopy is also being employed now as an alternative to confocal microscopy for 3D and deep tissue imaging, obviating the need for sectioning of 3D tissue-engineered constructs that sometimes can compromise the sample quality (Gioiella et al., 2016; Hume et al., 2018).

**Figure 7:**
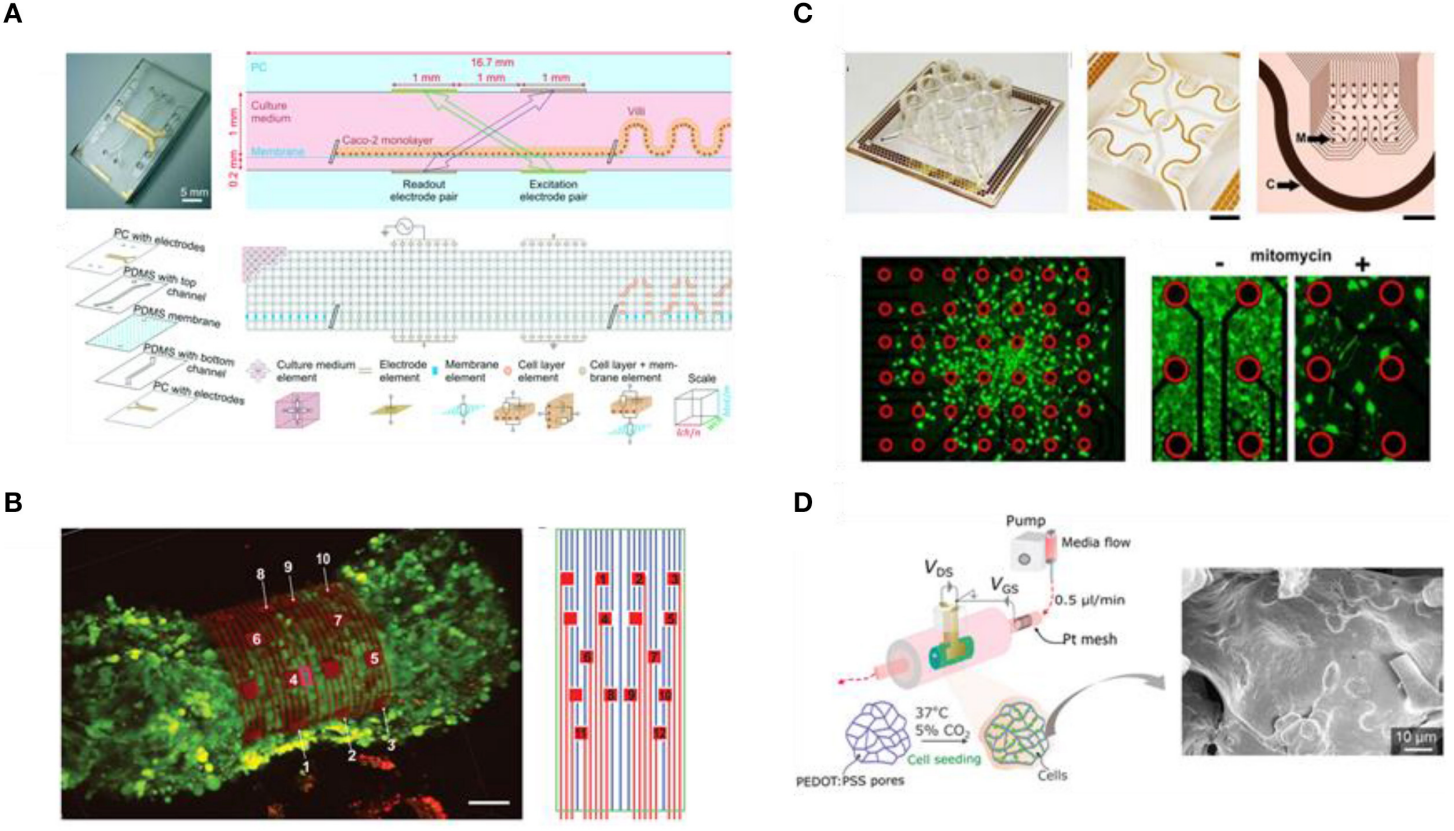
Biomimetic *in vitro* models of human tissues with integrated sensing and monitoring units. **(A)** Design and operation principles of a gut-on-chip device, along with the equivalent electrical circuit used for impedance monitoring of the intestinal barrier and the corresponding simulations. Reproduced from van der Helm et al. (2019) under the Creative Commons Attribution-Non Commercial License. **(B)** A 3D confocal microscopy image of 3D cardiac spheroid labeled with Ca^2+^ indicator dye (Fluo-4, green fluorescence) encapsulated by a self-roll biosensor array for electrical recordings; organ-on-e-chip (left) and 2D map of the microelectrodes of the biosensor array (right). Reproduced from Kalmykov et al. (2019) under the Creative Commons Attribution-Non Commercial license. **(C)** Image and design of a novel multi-well high-dense microelectrode array for cell migration studies (top), cell migration pattern for mitomycin C treated human breast cancer cells on the microelectrode array (bottom left), and comparative magnification to mitomycin C untreated cells (red circles mark electrodes) (bottom right). Adapted from Jahnke et al. (2019) under the Creative Commons Attribution International License. **(D)** Schematic representation of the setup of a 3D transistor in a tube (Tubistor), based on electroactive scaffolds (left), for hosting and concurrently monitoring 3D cell cultures. Reproduced from Pitsalidis et al. (2018) under the Creative Commons Attribution-Non Commercial license.

Besides new imaging assays, electrical transducers have been also shown to provide a wealth of real-time information through non-invasive and dynamic interfacing with biological systems (Rivnay et al., 2018). A well-established tool for rapid monitoring of cells *in vitro*, especially for drug toxicology studies, is Electrochemical Impedance Spectroscopy (EIS), with which it is possible to obtain information about cell adhesion, proliferation, and differentiation over time. In fact, EIS is widely used for monitoring Transepithelial/Transendothelial Electrical Resistance (TEER), a commonly used parameter to quantitatively characterize the function and integrity of tissue barriers with fast, non-invasive measurements (Benson et al., 2013; De León et al., 2020). van der Helm et al. recently developed an intestinal OOC with integrated electrodes that allowed for transepithelial barrier function and tissue differentiation monitoring via impedance spectroscopy, while combination with electrical simulation showed that this method can be adapted within any organ-on-chip to better monitor cell activity and to also enable comparisons between different platforms (van der Helm et al., 2019; Figure 7A). Integration of electrical transducers has also been reported recently in 3D biomimetic cultures (Pas et al., 2017; Zhang et al., 2017; Curto et al., 2018; Jahnke et al., 2019; Li et al., 2019). Kalmykov et al., for example, developed an “organ-on-e-chip” where they interfaced human cardiac spheroids with 3D self-roll biosensor arrays, which operated either as microelectrodes for EIS monitoring or as field-effect transistors, enabling acquisition of continuous multiplex recordings that allowed for real-time monitoring of cardiac tissue maturation (Kalmykov et al., 2019; Figure 7B). A high-density multi-electrode array was also proposed for real-time and automated impedimetric monitoring of cell migration out of human breast microtumours (Jahnke et al., 2019; Figure 7C). Despite the great progress made, most of these models fail to achieve intimate electrode-cell coupling which is necessary to accurately record a signal, since they utilize electrodes that are designed for planar culture of cells and are thus ill-adapted for monitoring complex 3D tissues (Inal et al., 2017; Jahnke et al., 2019). To overcome this limitation, conducting polymer scaffolds can be used instead, as evidenced recently by their remarkable performance as tissue building blocks (Wan et al., 2015; Guex et al., 2017; Iandolo et al., 2020). However, what makes these materials more attractive as TE substrates is that in addition to more seamless integration with complex cell cultures, they also allow for more intimate cell-electrode coupling, necessary for accurate signal transduction, and hence more effective monitoring of cell status and tissue formation (Inal et al., 2017; del Agua et al., 2018; Jayaram et al., 2019). We recently explored the potential of these materials for *in vitro* TE applications and organ-on-chip platforms. In particular, we fabricated tubular 3D macroporous electroactive scaffolds, based on the conducting polymer poly(3,4-ethylenedioxythiophene) doped with poly(styrene sulfonate) (PEDOT:PSS), the electrical, mechanical and biochemical properties of which we were able to fine-tune. We then integrated these tubular PEDOT:PSS scaffolds into a transistor configuration (i.e., transistor in a tube: Tubistor) and showed proof of principle for continuous monitoring of a simple 3D co-culture of mammalian cells over a period of 4 days. The real-time electrical readouts, cross-validated with optical analysis, enabled us to closely monitor cellular activity and even distinguish between cell adhesion and barrier tissue formation in a non-destructive, label-free manner, highlighting the added benefits of integrating in-line sensing components within engineered tissues for building more robust and sophisticated TE models (Pitsalidis et al., 2018; Figure 7D).

## Conclusions and Outlook

The implementation of TE concepts and methods into biomimetic tissue models, originally directed at regenerative medicine applications, is an accelerating trend. As summarized here, 3D *in vitro* models of various human tissues and organs have been successfully developed, thanks to the synergistic progress and advances in all disciplines that converge to give rise to more sophisticated tissue and disease models with enhanced structural and functional accuracy. Although many challenges are yet to be resolved, the advent of hiPSCs and organoids has, without a doubt, provided bioengineers with unlimited sources of tissue-specific cells, genetic engineering of which enables further modifications (e.g., insert/delete mutations in healthy/diseased cells) to guide phenotypic behaviors. This provides an excellent opportunity for transforming drug development routes by accelerating the process, and generating data more accurate and relevant to human systems. In addition, the use of patient-specific cells facilitates studies of rare diseases, as well as precision and personalized medicine approaches for the development of drugs and therapies optimized for specific patient biology. Smart biomaterials have now been designed to more faithfully recapitulate the chemical, mechanical and topographical properties of the complex human tissues and their microenvironment. Advanced fabrication methods have additionally enabled arrangement of tissue counterparts and environmental cues with unprecedented control and accuracy. These features have combined to generate complex biological structures of high fidelity, matching the *in vivo* situation. A key challenge remains however: monitoring and characterization of such models still relies predominantly on end-point, invasive assays, delaying the validation of results, particularly for drug screening, and toxicology studies. Integration of electrical components in tissue engineering platforms can help resolve this limitation. Such tools enable dynamic, non-invasive and continuous monitoring of cells, offering rapid insight into different biological events within these systems. Standardization of components such as media formulation is a non-trivial additional challenge, for maintaining the survival and function of multiple cell types, which combined with spatiotemporal delivery of tissue-specific and application-specific (i.e., homeostatic or pathophysiological conditions) environmental cues, offer a novel means for dynamically controlling and directing tissue generation and maturation *in vitro*. Without a doubt *in vitro* tissue engineered systems have shown great promise for revolutionizing many aspects of biomedical research. As more sophisticated and human relevant *in vitro* models appear in literature, in parallel with commercialization of platforms for hosting such models, we expect to see a paradigm shift in our understanding of human biology as well as in disease diagnosis and treatment.

## Author Contributions

C-MM and CB wrote the text and prepared the figures. RMO wrote the text and edited the manuscript and secured the funding. All authors contributed to the article and approved the submitted version.

## Conflict of Interest

The authors declare that the research was conducted in the absence of any commercial or financial relationships that could be construed as a potential conflict of interest.
